# Combining Evolutionary Information and an Iterative Sampling Strategy for Accurate Protein Structure Prediction

**DOI:** 10.1371/journal.pcbi.1004661

**Published:** 2015-12-29

**Authors:** Tatjana Braun, Julia Koehler Leman, Oliver F. Lange

**Affiliations:** 1 Biomolecular NMR and Munich Center for Integrated Protein Science, Department Chemie, Technische Universität München, Garching, Germany; 2 Department of Chemical and Biomolecular Engineering, Johns Hopkins University, Baltimore, Maryland, United States of America; Harvard Medical School, UNITED STATES

## Abstract

Recent work has shown that the accuracy of *ab initio* structure prediction can be significantly improved by integrating evolutionary information in form of intra-protein residue-residue contacts. Following this seminal result, much effort is put into the improvement of contact predictions. However, there is also a substantial need to develop structure prediction protocols tailored to the type of restraints gained by contact predictions. Here, we present a structure prediction protocol that combines evolutionary information with the resolution-adapted structural recombination approach of Rosetta, called RASREC. Compared to the classic Rosetta *ab initio* protocol, RASREC achieves improved sampling, better convergence and higher robustness against incorrect distance restraints, making it the ideal sampling strategy for the stated problem. To demonstrate the accuracy of our protocol, we tested the approach on a diverse set of 28 globular proteins. Our method is able to converge for 26 out of the 28 targets and improves the average TM-score of the entire benchmark set from 0.55 to 0.72 when compared to the top ranked models obtained by the EVFold web server using identical contact predictions. Using a smaller benchmark, we furthermore show that the prediction accuracy of our method is only slightly reduced when the contact prediction accuracy is comparatively low. This observation is of special interest for protein sequences that only have a limited number of homologs.

## Author Summary

Recently, a breakthrough has been achieved in modeling the atomic 3D structures of proteins from their sequence alone without requiring any experimental work on the protein itself. To achieve this goal, a database of evolutionary related sequences is analyzed to find co-evolving residues, giving insight into which residues are in close proximity to each other. These residue-residue contacts can help to drive a computer simulation with an atomic-scale physical model of the protein structure from a random starting conformation to a native-like 3D conformation. Although much effort is being put into the improvement of residue-residue contact predictions, their accuracy will always be limited. Therefore, structure prediction protocols with a high tolerance against incorrect distance restraints are needed. Here, we present a structure prediction protocol that combines evolutionary information with the iterative sampling approach of the molecular modeling suite Rosetta, called RASREC. RASREC has been shown to converge faster to near-native models and to be more robust against incorrect distance restraints than standard prediction protocols. It is therefore perfectly suited for restraints obtained from predicted residue-residue contacts with limited accuracy. We show that our protocol outperforms other currently published structure prediction methods and is able to achieve accurate structures, even if the accuracy of predicted contacts is low.

“This is a *PLOS Computational Biology* Methods paper”

## Introduction

The computational prediction of protein structures from their amino acid sequence is an ongoing challenge that has occupied scientists for more than four decades. While Anfinsen’s dogma [1] suggests that for most proteins the information contained in their amino acid sequence is sufficient to define their three-dimensional structure, the problem still remains largely unsolved. For some small proteins (<80 residues), current *ab initio* prediction methods are successful in predicting the corresponding 3D structures with high accuracy. One such method is the Rosetta *ab initio* protocol, which assembles short fragments of known proteins by a Monte Carlo strategy [2,3]. With increasing protein size however, sampling of the large conformational space becomes a major challenge [4] and combination with experimental data is required to achieve accurate protein models [5,6].

As experimental data is not always available and may be difficult or costly to obtain, researchers have focused on reducing the search space of possible protein conformations in other ways, for instance by including evolutionary information found in patterns of correlated mutations in protein sequences. The underlying assumption is that these correlated pairs indicate spatial proximity in the protein structure and can therefore be used to guide *ab initio* protein structure prediction [7].

The idea has already been introduced in the early 1990s [8–11], however, until recently, the accuracy of the predicted contacts was not sufficient to significantly improve structure prediction methods. Pairs of correlated mutations have been calculated using ‘local’ statistical models, e.g. mutual information scores, which are not able to separate direct from indirect contact information. While direct contacts reflect actual contacts in the protein structure, indirect contacts are false positives that arise from connections through a third residue. These transitive (indirect) pair correlations greatly limit the accuracy of predicted residue-residue contacts [7].

Recently, a substantial increase in prediction accuracy has been achieved by using ‘global’ statistical models [12–16] that are able to reduce these effects of transitivity by treating pairs of residues dependent on each other. Another important factor for the recent boost in prediction accuracy is the rapid growth of available protein sequences due to advances in DNA sequencing technology [7].

In 2011, it has been shown that the information contained in maximum-entropy derived residue-residue contacts is sufficient to predict protein folds with explicit atomic coordinates quite accurately (Cα-RMSDs of 2.7–4.8Å over at least two-thirds of the protein) using the method EVFold [13]. Since then, a lot of research focused on improving the contact predictions and new methods for residue-residue contact prediction emerge regularly [17–21]. In addition to the initial predictions of mostly soluble proteins [13], predicted contacts from evolutionary information have been used to predict protein-protein complexes [22–24], and the structures of membrane proteins [25,26].

While much effort is put into the improvement of contact predictions, there is also a substantial need to investigate how this information is best exploited in structure prediction. The accuracy of contact predictions is limited by the statistical nature of the prediction methods, distracting sources of co-evolution (e.g. active sites and protein-protein interaction sites), and limited numbers of homologous sequences. Due to the noisy nature of the predicted residue-residue contacts, structure prediction protocols with a high tolerance against incorrect distance restraints are needed.

EVFold uses the CNS molecular dynamics software suite [27,28] for structure prediction. It starts with the fully extended amino-acid sequence and folds the protein by applying standard distance geometry techniques and simulated annealing with bonded and non-bonded potentials [13].

The fragment-based folding algorithm FRAGFOLD [29,30] was used in combination with the contact prediction method PSICOV [17] for *ab initio* structure prediction [31]. The restraints were scored with a square well function with exponential decay.

Michel and coworkers applied the *ab initio* structure prediction protocol of the molecular modeling software suite Rosetta [32] with a smoothed square well restraint scoring function to predict structures within the PconsFold pipeline [33]. A comparison between Rosetta and CNS indicated that with similar contact predictions, models of similar quality were generated [33]. Improvements in structure prediction were mainly credited to improved residue-residue contact predictions obtained with the combined prediction method PconsC [34].

The CONFOLD webserver uses the CNS suite [27,28] for a two-stage modeling approach. Both, restraints derived from predicted contacts and secondary structure, are used and after the initial round of model generation, unsatisfied restraints are filtered out. The method has been shown to be especially powerful when using true contacts [35].

**Fig 1:**
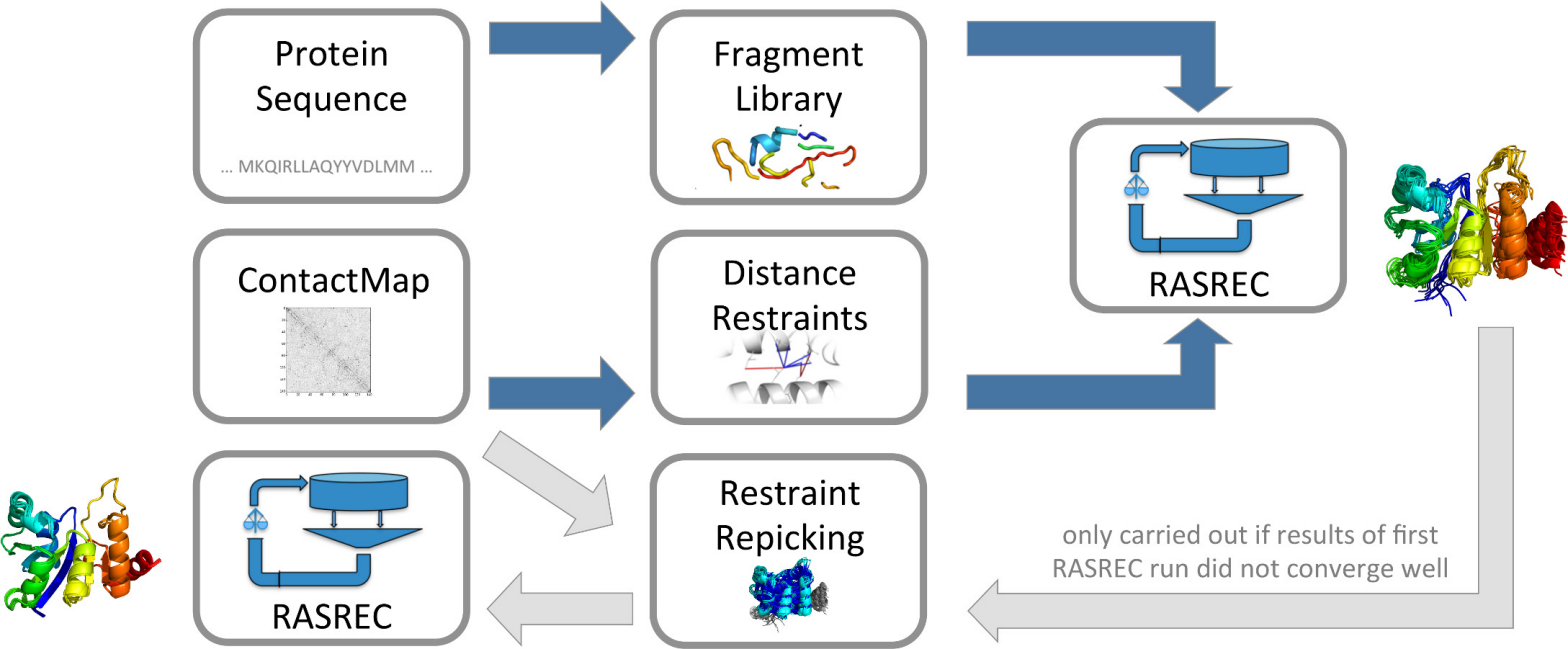
Protocol pipeline. Our protocol consists of one core step (blue) and an optional refinement step (light grey). Core step: The top scoring residue pairs of a predicted contact map are translated into distance restraints and used for structure prediction in combination with the RASREC protocol. Refinement step: Restraints are repicked from the results of the core step and used in a second RASREC run combined with additional contact map restraints.

In this work we combine evolutionary information, obtained from predicted residue-residue contacts, with the Resolution-Adapted Structural RECombination approach RASREC [36] (cf. Fig 1). RASREC is an iterative sampling protocol of Rosetta that carries out restraint-guided fragment assembly during six different resampling and refinement stages. The main idea behind the protocol is the iterative recombination of frequently reoccurring structural features and promising strand pairings. It has been shown previously that RASREC requires less data and is more robust against incorrect distance restraints than the standard Rosetta prediction protocol [5,6,36]. These properties make RASREC the ideal starting point for developing a protocol for structure prediction guided by evolutionary restraints, the latter containing a fraction of incorrectly predicted protein-protein contacts.

For our method, evolutionary information was added to the RASREC protocol by translating the top scoring residue-residue contact pairs into sigmoidal distance restraints. This initial RASREC prediction was furthermore followed by an additional refinement run using distance information from both the previous run and the predicted residue-residue contacts.

To investigate the performance of our method, we carried out a benchmark on 28 globular proteins using state-of the-art contact predictions (generated using a pseudo-likelihood maximization approach). To test the impact of increasing numbers of false restraints, we additionally predicted the structures of a smaller benchmark set using less accurate residue-residue contact predictions (calculated with a mean-field direct coupling analysis).

In this manuscript we report the results of the benchmark using both types of residue-residue contact predictions and contrast the performance of our protocol with results obtained by the EVFold webserver using identical contact predictions. We furthermore illustrate the contribution of the optional refinement run to the final results of our method and investigate the benefits of including predicted residue-residue contacts to the standard RASREC sampling method in general.

## Materials and Methods

### Datasets

We have benchmarked our protocol on two previously published datasets, namely the 14 globular proteins from the EVFold benchmark set published in [13] and the 14 globular proteins used as test set for developing Pconsfold [33]. The structures vary in sequence length between 58 and 247 residues and cover the three structured CATH classes i.e. mainly α, mainly β, and mixed α/β. An overview of all targets in our benchmark set can be found in Table 1.

**Table 1:** Benchmark set. Positive predictive values (PPV) have been calculated for two restraint sets (calculated with the pseudo-likelihood maximization approach (PLM) and direct coupling analysis (DI), respectively) by comparing the potential contacts to the actual Cβ-Cβ distances in the reference structure with a cutoff of 8 Å.

Benchmark set	Target	Fold (CATH)	Model Size	# Restraints	PPV Distance Restraints	
					PLM	DI
**EVFold benchmark set**	2hda	β	58	50	0.78	0.52
	5pti	few ss	63	60	0.67	0.65
	1wvn	α/β	73	70	0.64	0.39
	1g2e	α/β	81	80	0.84	0.65
	1odd	α	87	80	0.54	0.28
	1rqm	α/β	105	100	0.61	0.55
	1r9h	α/β	105	100	0.79	0.64
	2o72	β	110	110	0.76	0.65
	1bkr	α	117	110	0.45	0.33
	2it6	α/β	117	110	0.68	0.49
	1e6k	α/β	124	120	0.73	0.61
	1f21	α/β	147	140	0.69	0.44
	5p21	α/β	170	170	0.48	0.48
	3tgi	β	226	220	0.79	0.50
**Pconsfold benchmark set**	1jo8	β	58	50	0.80	-
	1bdo	β	80	80	0.51	-
	1fqt	β	112	110	0.85	-
	2cua	β	135	130	0.57	-
	1vp6	β	138	130	0.66	-
	1a3a	α/β	148	140	0.79	-
	1ihz	β	149	140	0.78	-
	1jwq	α/β	179	180	0.65	-
	1im5	α/β	180	180	0.72	-
	1atz	α/β	189	180	0.81	-
	1chd	α/β	203	200	0.81	-
	1hdo	α/β	206	200	0.43	-
	1o1z	α/β	234	230	0.71	-
	1tqh	α/β	247	240	0.68	-

In case of the EVFold benchmark set, the protein sequences of the models published in [13] (available at http://evfold.org/evfold-web/datasets.do) were used to enable a direct comparison between EVFold and our method. For the Pconsfold dataset, the sequences deposited in the RCSB Protein Data Bank [37] were used. FASTA sequences for all targets in our benchmark set are available in S2 File.

### Contact prediction

We used two sets of contact predictions, generated with the PLM (pseudo-likelihood maximization) and DI (direct information/ mean field approximation) scoring method, respectively.

The PLM method uses a pseudo-likelihood maximization approach [19,38] for finding the maximum entropy set of correlated interactions. This approach is one of the most accurate prediction methods to date [20]. Residue contacts based on this scoring method were predicted for the entire benchmark set using the EVFold webserver (available at http://www.evfold.org/) with default parameters. EVFold returns, along with the predicted 3D models, a list of all-by-all residue pairings computed with EVcouplings-PLM. Restraints based on these contact predictions will be referred to as PLM-restraints in the remainder of this manuscript.

The DI method, as published in [13], uses a less accurate mean field approximation. The contact predictions used in [13] are provided as downloadable content on the EVFold website. Restraints extracted from these contact predictions will be referred to as DI-restraints in the remainder of this manuscript.

In EVFold, contact predictions are further processed by applying several filters based on residue conservation, secondary structure prediction and cysteine pairings [13] before being translated to distance constraints. In contrast, we used the predicted contacts without any filters to see how much information they provide by themselves. For both restraint sets, the predicted contacts were ordered by their assigned confidence score and the *L* top-ranked contacts with a minimum distance of 5 residues were selected (with *L* being the length of the protein sequence rounded down to the nearest multiple of 10). Unless mentioned otherwise, predicted residue contacts refer to these *L* top-ranked contacts.

The accuracy of the contact predictions was assessed in form of the positive predictive value (PPV) by comparing a potential contact to the actual Cβ-Cβ distance in the reference structure. A contact was counted as a true positive if the Cβ-Cβ distance in the native structure is ≤ 8 Å.

### Structure generation with RASREC

To generate the three-dimensional structures, we used the RASREC protocol as described previously [36]. For objective benchmarking and mimicking real application cases, homologous structures (with a PSI-BLAST [39] e-score < 0.05) were excluded in creating the fragment library of each target.

Instead of using experimentally derived distance restraints, we used the predicted residue contacts as source of residue-residue distance information. For this purpose, the *L* top scoring contact predictions were translated into Rosetta specific Cβ-Cβ distance restraints as described below.

To account for the fact that the predicted contacts might be noisy and might contain a varying number of incorrectly predicted contacts (i.e. false positives), the distance restraints were scored with a shallow sigmoidal potential [23]:

$$f_{\text{Sigmoid}}\left(x\right)=\frac{1}{1+e^{-m\cdot \left(x-x_{0}\right)}}-0.5\;\text{with}\;x_{0}=8.0\;\text{and}\;m=1$$ (1)

Satisfied distance restraints (Cβ-Cβ distance ≤ 8 Å) add a bonus to the final energy term, while unsatisfied distance restraints are ignored. This greatly reduces the influence of incorrectly predicted residue contacts and the structure prediction will not be misguided. Using bounded restraints in this step instead, i.e. punishing each violated restraint with an energy penalty, often resulted in misfolded and unconverged structures in initial test runs.

As in [36], the pool size of RASREC, specifying the number of best scoring models maintained during each iteration stage, was set to 500. The total number of models generated during a RASREC run depends on how fast the different iteration stages terminate and cannot be directly controlled. For the EVFold benchmark set, the total number of generated models per target ranges from 13,000 to 65,000. For a detailed description of all options and parameters used, please refer to S1 Supporting Information and the Protocol Capture in S1 Text and S1 File.

RASREC requires substantial computer resources. For the EVFold benchmark set, the average computation time was ~2600 cpu hours using 2.6 GHz AMD Opteron processors, see Fig A in S1 Supporting Information. The computation time is dependent on several factors, which include sequence length, fold complexity, and instructiveness of the restraints.

#### Optional refinement step

If the results of the first RASREC run did not converge in all parts of the protein structure (fraction of converged residues < 90% in the 30 lowest energy models), an optional refinement run (ReRASREC) was carried out to increase both accuracy and convergence. For this purpose, converged substructures from the initial RASREC run were rebuilt and non-converged regions were refined using additional contact information:

To easily re-establish the converged core of the initial RASREC run, we derived distance restraints for the converged regions in the following way: Distances between all Cα-Cα pairs were calculated, and those that are short-range (≤ 8Å) and have a standard deviation (SD) below 1Å in the 30 low-energy RASREC models were kept. These converged distances were enforced during ReRASREC using the strict bounded potential as in [6]:

$$f_{\text{Bounded}}\left(x\right)=\left\{ \begin{array}{l} \;{\left(\frac{x-lb}{sd}\right)}^{2}\;\text{for}\;x<lb\\ \;0\;\text{for}\;lb\leq x\leq ub\\ \;{\left(\frac{x-ub}{sd}\right)}^{2}\;\text{for}\;ub<x\leq ub+0.5*sd\\ \frac{1}{sd}\left(x-\left(ub+0.5*sd\right)\right)+{\left(\frac{0.5*sd}{sd}\right)}^{2}\;\text{for}\;x>ub+0.5*sd \end{array}\right.\text{with}\;sd=1$$ (2)

To reflect the average distance *d* in the converged region, the lower bound *lb* was set to *(d*– 1) and the upper bound *ub* to (*d+1*).

The structural models from the first RASREC run allowed us to select additional low-ranked predictions from the contact map: Prior to having any structural knowledge we could only choose contact predictions with very high confidence in the attempt to avoid frustrating the calculations with too many erroneous restraints. In the second iteration however, we were able to use the lowest-energy models of the first RASREC run to filter out contact predictions that clearly disagree with these models. Hence, lower-confidence predictions could be incorporated as well. To refine the unconverged regions (residue-residue distance, SD > 1 Å in 30 low-energy structures), we therefore chose additional residue-residue pairings from the predicted contact map that affect these regions and do not totally disagree (i.e. are short range with an average distance d ≤ 8 Å) with the lowest-energy models of the first run. The restraints were scored with a wide bounded potential with lower and upper bound set to 1.5 Å and 8 Å, respectively. This wide range was chosen to allow these regions to adapt to energetically favorable conformations. To reduce the influence of potentially incorrect restraints in this set, we furthermore combined random pairs into ambiguous restraints [6]. For each model new random pairs were generated.

#### Identifying unsuccessful predictions by backbone convergence

For “blind” structure predictions it is important to discern whether the final result of a prediction method is reliable or not. Here, we used the backbone convergence of the 30 lowest-energy models as a criterion to decide whether a prediction is classified as successful or not. The backbone of a residue was considered converged if the corresponding Cα-atoms in the 30 lowest-energy structures had less than 2 Å coordinate variability. If less than half of the residues of the 30 lowest-energy structures converged, a prediction was regarded as unsuccessful. In those cases, our protocol was not able to find a consistent low energy state.

#### Model ranking

The models predicted by RASREC were ranked according to their resulting Rosetta Energy Units (REU). Distance restraints were included with a weight of 0.1 in this full-atom energy function. The ensemble of the 10 lowest-energy structures is considered as the final result of our protocol. Therefore, if not stated otherwise, the metrics used for performance evaluation are averaged over the 10 lowest-energy structures.

### Structure prediction with EVFold

The EVFold webserver offers to directly fold the protein of interest based on its predicted residue-residue contacts. Structure prediction is accomplished using the CNS software [27,28] with the protocol described in [13]. The webserver predicts structures for different amounts of filtered restraints, starting with only a few and increasing to *L* in 10 steps with *L* being the domain length. As output, the 3D coordinates of all 50 predicted structures are provided. We used the web interface to generate the models along with the predictions based on the PLM approach. These models are referred to as EVFold-PLM models. Further, we used the structures published in [13] (available at http://evfold.org/evfold-web/datasets.do), which are based on the residue-residue contact predictions with the less accurate DI approach and are referred to as EVFold-DI models.

#### Model ranking

EVFold ranks its models with a score based on inherent properties and extent of constraint satisfaction. We consider the single top-ranked structure as the final result of EVFold, irrespective of the number of distance restraints used. In addition, results averaged over the 10 top-ranked structures can be found in Table C in S1 Supporting Information.

### Metrics used for performance evaluation

To evaluate the performance of our method, several different metrics were used: 1) Cα-RMSD calculated over all residues present in the reference structure (RMSD), 2) Cα-RMSD calculated over all residues in secondary structural elements in the crystal structure as assigned by Stride [40] called RMSD_SSE_, and 3) TM-Score [41] over all Cα-atoms in the reference structure. The template modeling score (TM-Score) evaluates the global fold similarity and is less sensitive to local structural variations than the RMSD. It ranges from 0 (random similarity) to 1 (perfect similarity) [41].

In contrast to e.g. RMSD values calculated with PyMOL [42], which excludes outliers in a series of refinement cycles, these three metrics are easily reproducible and consider the same residues for each model evaluated.

## Results and Discussion

We have developed a protocol (Fig 1) that combines RASREC with evolutionary sequence information in form of predicted residue-residue contacts for accurate protein structure prediction. We benchmarked this protocol on a diverse set of 28 globular proteins and compared its results with the ones from the EVFold web server, to our knowledge one of the best methods currently available.

### Models generated with ReRASREC have higher accuracies

**Fig 2:**
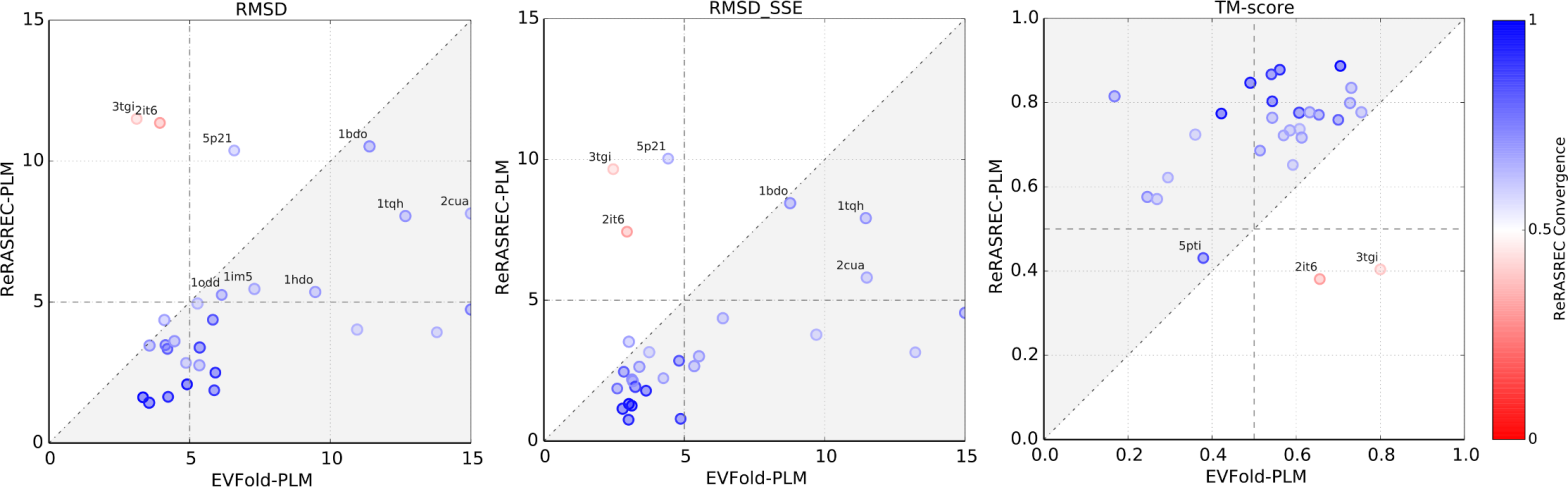
Comparison between ReRASREC-PLM and EVFold-PLM. In case of ReRASREC-PLM, the similarity measures are averaged over the 10 lowest-energy models, while for EVFold-PLM the single top ranked model is evaluated. The color represents the fraction of converged residues in the 30 lowest-energy models of ReRASREC-PLM. The gray areas indicate an improvement of ReRASREC-PLM over EVFold-PLM.

Fig 2 shows the performance of our protocol (ReRASREC-PLM) compared to the one of the EVFold web server (EVFold-PLM) on the basis of three different metrics. Our protocol converged (fraction of converged residues > 0.5 in the 30 low-energy structures) for 26 out of the 28 targets and correctly predicted the fold for each of the converged targets (TMscore > 0.5 or RMSD < 5Å). For the majority of the benchmark set, the final models were of high structural accuracy resulting in an average TM-score of 0.74, an average RMSD of 4.4 Å, and an average RMSD_SSE_ of 3.3 Å over all 26 converged targets.

The overall performance of our protocol was significantly higher than that of EVFold-PLM using identical contact predictions (however not necessarily identical distance restraints, see section Structure Prediction with EVFold). With an average TM-score of 0.72 over the entire benchmark set, ReRASREC-PLM lead to an improvement of 0.17 when compared to EVFold-PLM, whose average TM-score was only 0.55. ReRASREC-PLM furthermore increased the number of targets with a TM-score > 0.7 from 6 to 20. In terms of RMSD and RMSD_SSE_, using our method lead to an average improvement from 7.3 Å to 4.9 Å and from 5.7 Å to 3.7 Å respectively. Moreover, EVFold-PLM failed to predict the correct fold for 6 out of 28 targets (TM-score < 0.5 and RMSD > 5Å) while our protocol predicted very accurate models (TM-Score 0.62) with correct folds for all of these targets.

Based on our backbone convergence criteria (see Materials and Methods) our protocol failed for targets 2it6 and 3tgi. Both targets consist of long loop regions (fraction of secondary structural content is only 0.54 and 0.37 respectively) and are therefore challenging for RASREC as it is mainly focusing on the recombination of reoccurring structural features such as secondary structure elements.

Fig 2 reveals that predictions for two converged targets, namely 5p21 and 1bdo, resulted in models with an RMSD > 10 Å. The TM-Score is however above 0.5 in both cases, i.e. 0.65 and 0.58, respectively, showing that the majority of the protein structure was predicted correctly. The good accordance between the top-scoring models and the corresponding native structures can furthermore be seen in Fig B in S1 Supporting Information.

ReRASREC-PLM was not only able to predict the correct fold for a larger number of targets, but also significantly improved the accuracy within the set of targets with correctly predicted folds. Excluding the 8 targets where either EVFold-PLM (6) or RASREC-PLM (2) had difficulties, ReRASREC-PLM still increased the average TM-Score by 0.18 over EVFold-PLM from 0.60 to 0.78. In terms of RMSD and RMSD_SSE_, RASREC-PLM improved them from 5.6 Å to 3.9 Å and from 4.2 Å to 2.9 Å, respectively.

We also compared the accuracy of ReRASREC-PLM with two other recently published methods (PconsFold [33] and FRAGFOLD [31]) on the subset of targets where each publication reported actual numbers on. We found that, although both methods improve upon EVFold-PLM, ReRASREC-PLM still outperforms both (Table A in S1 Supporting Information).

### ReRASREC models have accurate side chains in the protein core

**Fig 3:**
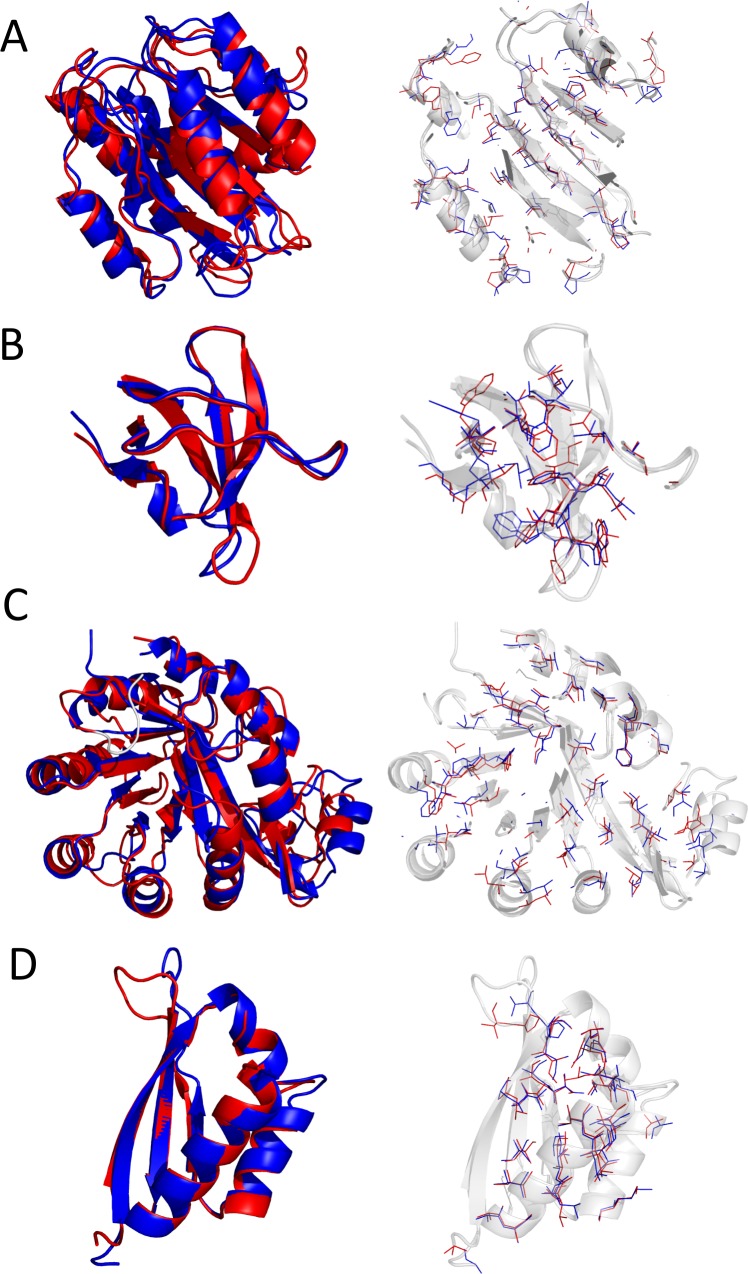
Superposition of top ranked models and corresponding crystal structures. Top-energy ReRASREC structures (red) for 1atz (A), 1jo8 (B), 1o1z(C), and 1wvn(D) are superimposed with the corresponding crystal structures (blue). For each target, a cartoon representation of the lowest-energy structure (left) and a close-up showing non-polar side-chains (right) is shown.

Fig 3 further indicates that the models generated with our protocol do not only have high accuracy in their backbones, but also a high rotamer recovery of core side-chain conformations. A superposition of the lowest-energy model and the corresponding crystal structure of each target can be found in Fig B in S1 Supporting Information.

Table 2 shows that on average 84% of the converged core side chains in the RASREC models are in the same χ_1_ rotamer well, and 46% have the same set of rotamer states for all χ angles as the corresponding crystal structures. An analysis of the single top-ranked models of EVFold-PLM and ReRASREC-PLM furthermore shows that ReRASREC-PLM predicts higher numbers of buried side chains with native χ_1_ romater assignment than EVFold-PLM, see Table B in S1 Supporting Information.

**Table 2:** Accuracy of sidechain χ1 rotamers in the final ReRASREC models. Buried and converged side chains are selected and their adopted rotamer assignments are compared to those in the reference crystal structure. Alanine and Glycine are excluded from this analysis.

Benchmark set	Target	Number of side chains			Fraction of recovered rotamers	
		buried*	converged & buried**	recovered χ1 ***	χ1 only^%^	all χ angles^~^
**EVFold benchmark set**	1bkr	42	8	7	0.88	0.50
	1e6k	49	20	18	0.90	0.55
	1f21	53	20	19	0.95	0.45
	1g2e	25	11	10	0.91	0.64
	1odd	27	10	9	0.90	0.70
	1r9h	36	8	7	0.88	0.63
	1rqm	42	12	7	0.58	0.33
	1wvn	19	14	13	0.93	0.50
	2hda	16	11	6	0.55	0.27
	2it6	48	7	6	0.86	0.29
	2o72	27	8	7	0.88	0.38
	3tgi	101	27	22	0.81	0.37
	5p21	71	20	19	0.95	0.60
	5pti	14	5	3	0.60	0.20
**Pconsfold benchmark set**	1a3a	56	19	16	0.84	0.63
	1atz	72	9	8	0.89	0.33
	1bdo	25	10	8	0.80	0.70
	1chd	74	23	19	0.83	0.43
	1fqt	44	21	19	0.90	0.52
	1hdo	84	21	15	0.71	0.38
	1ihz	51	7	6	0.86	0.29
	1im5	68	22	19	0.86	0.32
	1jo8	15	10	8	0.80	0.50
	1jwq	76	17	15	0.88	0.53
	1o1z	99	29	26	0.90	0.34
	1tqh	106	36	32	0.89	0.53
	1vp6	50	19	18	0.95	0.63
	2cua	46	15	11	0.73	0.27
**Average**	N/A	N/A	N/A	N/A	0.84	0.46

* Side chains that are buried in the reference structure (SASA < 40Å)

** Side chains that are buried (SASA < 40Å) and converged (χ1 angle, SD < 10 degrees in 10 low-energy structures).

*** Subset of converged and buried residues that adopt the same χ1 rotamer state as in the reference structure.

% Ratio of column 2 (correct) and column 1 (converged and buried)

~ Fraction of sidechains in column 1 (converged and buried) for which all side-chain torsion angles adopt the same rotamer state as in the reference structure.

### ReRASREC is more robust against incorrect distance restraints

It has been shown previously [5,6,36] that RASREC is more robust against incorrect distance restraints than the standard Rosetta *ab initio* protocol. A high tolerance against false positives is of special interest for proteins where only a limited number of homologous sequences are available. In those cases, the fraction of false positives in the corresponding contact predictions is comparably high, hence making structure prediction for standard prediction methods difficult.

To investigate how our protocol performs with an elevated amount of incorrectly predicted residue contacts, we used it in combination with the contact predictions published in [13]. These predictions were generated with the less accurate mean field approach (DI–direct information) and therefore contain an increased number of incorrectly predicted protein contacts as compared to the restraints obtained with the PLM approach (see Table 1). With an average PPV of 0.51, the accuracy of the DI-restraints drops by 0.17 compared to the average PPV of the PLM-restraints.

Given these restraints with a significantly lower accuracy, our protocol was able to converge for 12 out of 14 targets (see Fig C in S1 Supporting Information) and predicted the correct fold for all of the converged targets with an average TM-score of 0.70 and an average RMSD of 4.0 Å (see Table 3). The results obtained with our protocol significantly outperform the top ranked results generated with EVFold using DI-restraints: Using our protocol lead to an increase in average TM-score of 0.17 when compared to the average TM-score of 0.47 of the corresponding EVFold results. In terms of RMSD, the use of ReRASREC-DI improved the prediction from 7.2 Å to 5.6 Å. For 6 targets, the top-ranked EVFold models furthermore displayed the incorrect fold (TM-score < 0.5 and RMSD > 5 Å).

**Table 3:** Results for the EVFold benchmark set using different methods and different restraint sets. For ReRASREC, the metrics were calculated and averaged over the 10 lowest-energy models while for EVFold, the single top ranked structure was used. For both methods, results generated with both PLM- and DI-restraints are shown. For each double column and target, the ‘better’ performance is highlighted.

	TM-score				RMSD			
	PLM-restraints		DI-restraints		PLM-restraints		DI-restraints	
Target	ReRASREC-PLM	EVFold-PLM	ReRASREC-DI	EVFold-DI	ReRASREC-PLM	EVFold-PLM	ReRASREC-DI	EVFold-DI
1bkr	**0.62**	0.30	**0.68**	0.29	**3.93**	13.79	**3.67**	13.20
1e6k	**0.89**	0.71	**0.87**	0.63	**1.62**	3.34	**1.78**	4.76
1f21	**0.76**	0.70	**0.59**	0.51	**3.34**	4.21	**6.87**	8.16
1g2e	**0.88**	0.56	**0.84**	0.54	**1.64**	4.23	**1.83**	5.23
1odd	**0.69**	0.51	**0.49**	0.37	**5.26**	6.14	**6.20**	9.40
1r9h	**0.72**	0.57	**0.68**	0.48	**2.84**	4.87	**5.47**	7.19
1rqm	**0.80**	0.54	**0.78**	0.55	**2.50**	5.91	**2.46**	4.72
1wvn	**0.87**	0.54	**0.82**	0.28	**1.87**	5.87	**2.09**	8.21
2hda	**0.77**	0.42	**0.72**	0.40	**2.08**	4.91	**2.47**	6.59
2it6*	0.38	**0.66**	0.38	**0.39**	11.36	**3.94**	10.62	**10.54**
2o72	**0.77**	0.65	**0.69**	0.54	**3.48**	4.14	**4.41**	6.07
3tgi*	0.40	**0.80**	0.19	**0.53**	11.50	**3.12**	20.19	**7.66**
5p21	**0.65**	0.59	0.66	**0.70**	10.38	**6.58**	7.99	**3.64**
5pti	**0.43**	0.38	**0.62**	0.45	**4.37**	5.82	**2.77**	4.75
Mean	**0.69**	0.57	**0.64**	0.47	**4.73**	5.49	**5.63**	7.15

* unconverged targets

Using the less accurate DI-restraints had less of an impact on accuracy for ReRASREC than for EVFold; the average TM-score of the EVFold benchmark set decreased by 0.05 and by 0.1 points for ReRASREC and EVFold, respectively (Table 3). While ReRASREC predicted the correct fold for all 12 converged targets with both restraint sets, EVFold increased the number of incorrect folds from 2 to 6 when using the less accurate DI-restraints instead of PLM-restraints.

This suggests that our protocol can predict structures with restraints of mediocre accuracy better than the CNS protocol used by EVFold.

### Successful model ranking with full-atom energy function

For realistic application cases the ranking of the predicted structural models is of great importance as it will be the single criterion for selecting the final predicted models. The models generated with our protocol were ranked with the full-atom energy function of Rosetta. All-atom energy functions are very sensitive to correct packing of side chains due to the steep gradient of the Lennard-Jones repulsive term. Correct packing of side chains is hard to achieve, in particular, if the backbone structure is not sufficiently accurate. Selection based on this energy function is therefore only possible if the backbone accuracy is very high.

**Fig 4:**
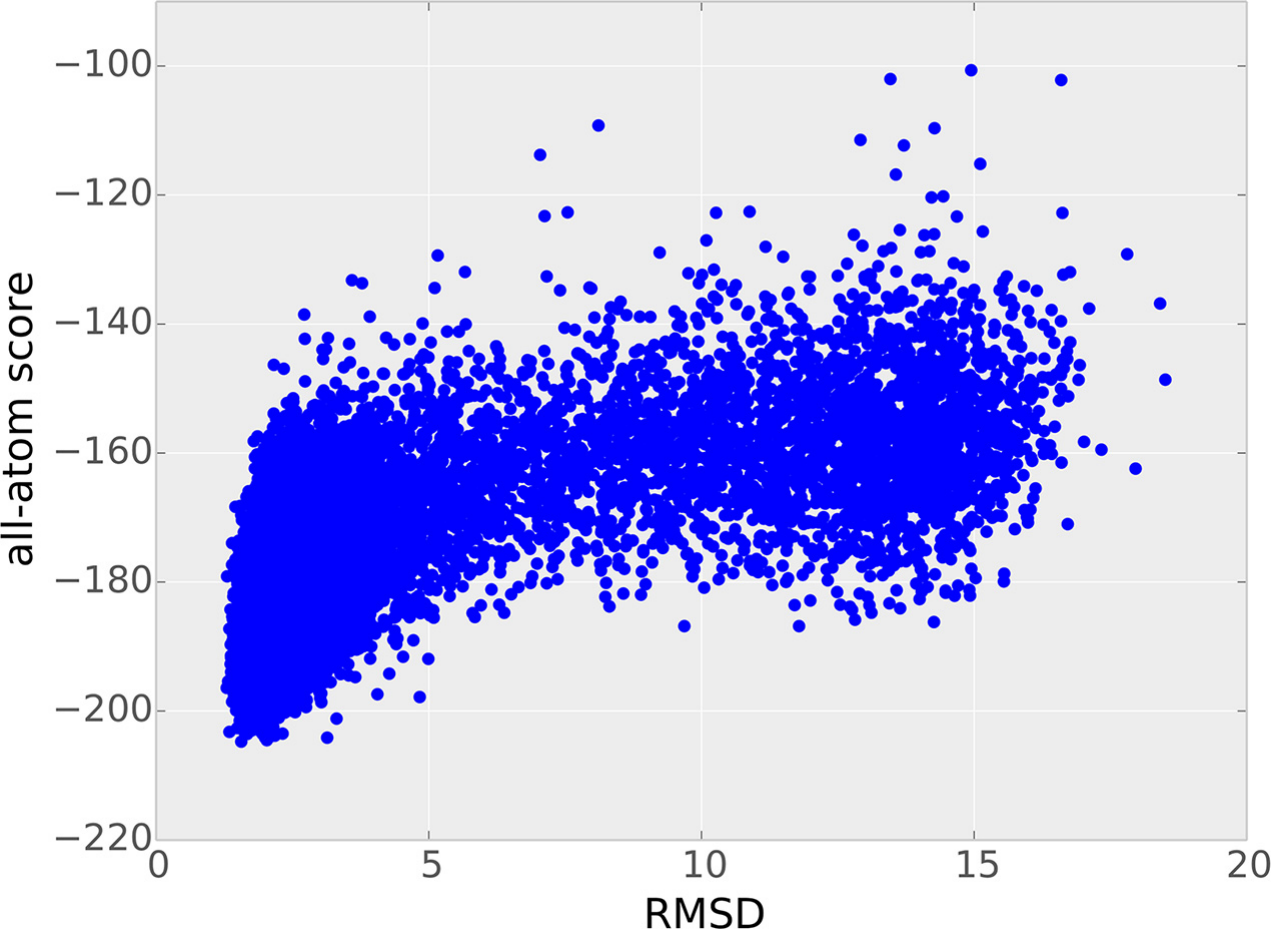
RMSDs and all-atom scores of each structure generated during a single RASREC run. All structures generated during the initial RASREC run of target 1e6k are shown. A simple structural refinement was carried out for each model to convert the centroid models (the first four RASREC stages use the Rosetta low-resolution energy) into full atom models with packed side chains.

Fig 4 shows the full-atom energies and RMSD values for each model generated during the different stages of a single RASREC run for one exemplary target. The energy funnel at the low RMSD area shows that the all-atom energy function is able to discriminate between correct and incorrect structural models.

This observation is further reinforced by comparing the lowest-RMSD models to the lowest-energy models (Table C in S1 Supporting Information): The average TM-score of the lowest-RMSD models is with 0.77 only 0.05 higher than the one of the lowest-energy models generated by ReRASREC with 0.72.

In contrast, EVFold ranks its models based on inherent geometrical properties and constraint satisfaction. Choosing the lowest-RMSD models instead of the top ranked ones increases the average TM-score from 0.55 to 0.62 and improves the RMSD from 7.3 Å to 5.2 Å.

Investigating these results more closely, one can observe that the top ranked structures of EVFold-PLM adapt the incorrect fold (RMSD > 5 Å and TM-score < 0.5) for two targets, namely 1bkr and 1o1z, although models with correct topologies were generated as well. For those two targets, the ranking of EVFold-PLM therefore fails. For ReRASREC-PLM using the full-atom score function, no such discrepancy was observed.

### Gain in accuracy due to high quality structural models

**Fig 5:**
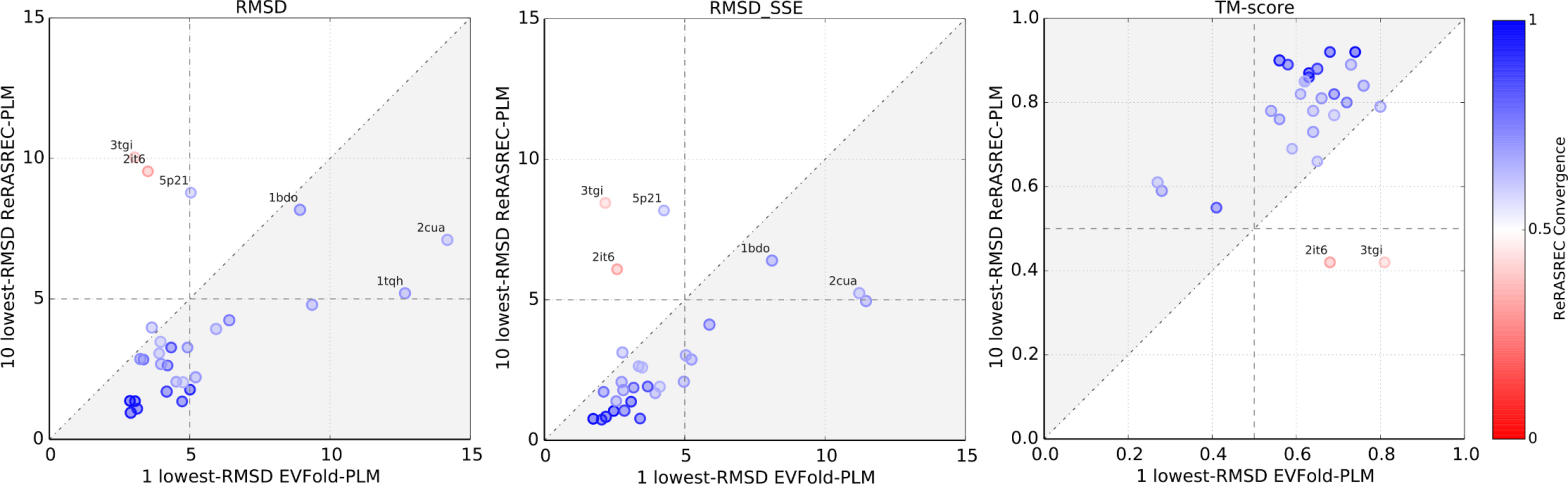
Comparison of ReRASREC’s lowest-RMSD models to the lowest-RMSD models generated with EVFold. The single most accurate EVFold structure (lowest RMSD) has been selected among all 50 provided models and is compared to the average of the 10 models of a RASREC refinement run with lowest RMSD.The color represents the fraction of converged residues in the 30 lowest energy models of ReRASREC-PLM. Gray shaded areas indicate an improvement of ReRASREC-PLM over EVFold-PLM.

In this section, we analyze the accuracy of the models generated by EVFold-PLM and ReRASREC-PLM irrespective of their ranking schemes. Therefore, we have compared the most accurate models (average of the 10 lowest-RMSD models) of ReRASREC to the single lowest-RMSD models generated by the EVFold web server within its 50 reported models. As shown in Fig 5, the ReRASREC models with lowest RMSD outperform the lowest-RMSD models of EVFold for each converged target. Overall, the ReRASREC models show an increase in TM-score of 0.15 when compared to the average TM-score of 0.62 of the single most accurate EVFold models.

We have shown in the previous section that the difference in accuracy between the lowest-energy and lowest-RMSD models of ReRASREC-PLM is small. The lowest energy models of ReRASREC-PLM are therefore more accurate than any models obtained with the EVFold webserver (see Fig D in S1 Supporting Information). On average, the lowest-energy models of ReRASREC-PLM lead to an increase in TM-score of 0.1 when compared to the TM-score of 0.62 of the single lowest-RMSD models of EVFold-PLM. This shows that our method generates models of higher structural quality than EVFold-PLM.

### Refinement run leads to small improvements in model accuracy

If the backbone of the first RASREC run did not converge within 2 Å for over 90 percent of the residues, a refinement run (see Materials and Methods) was carried out. To see to what extent the refinement run contributes to the final performance of our protocol, we compared the results of the initial RASREC run to the results obtained after the refinement run (ReRASREC).

**Fig 6:**
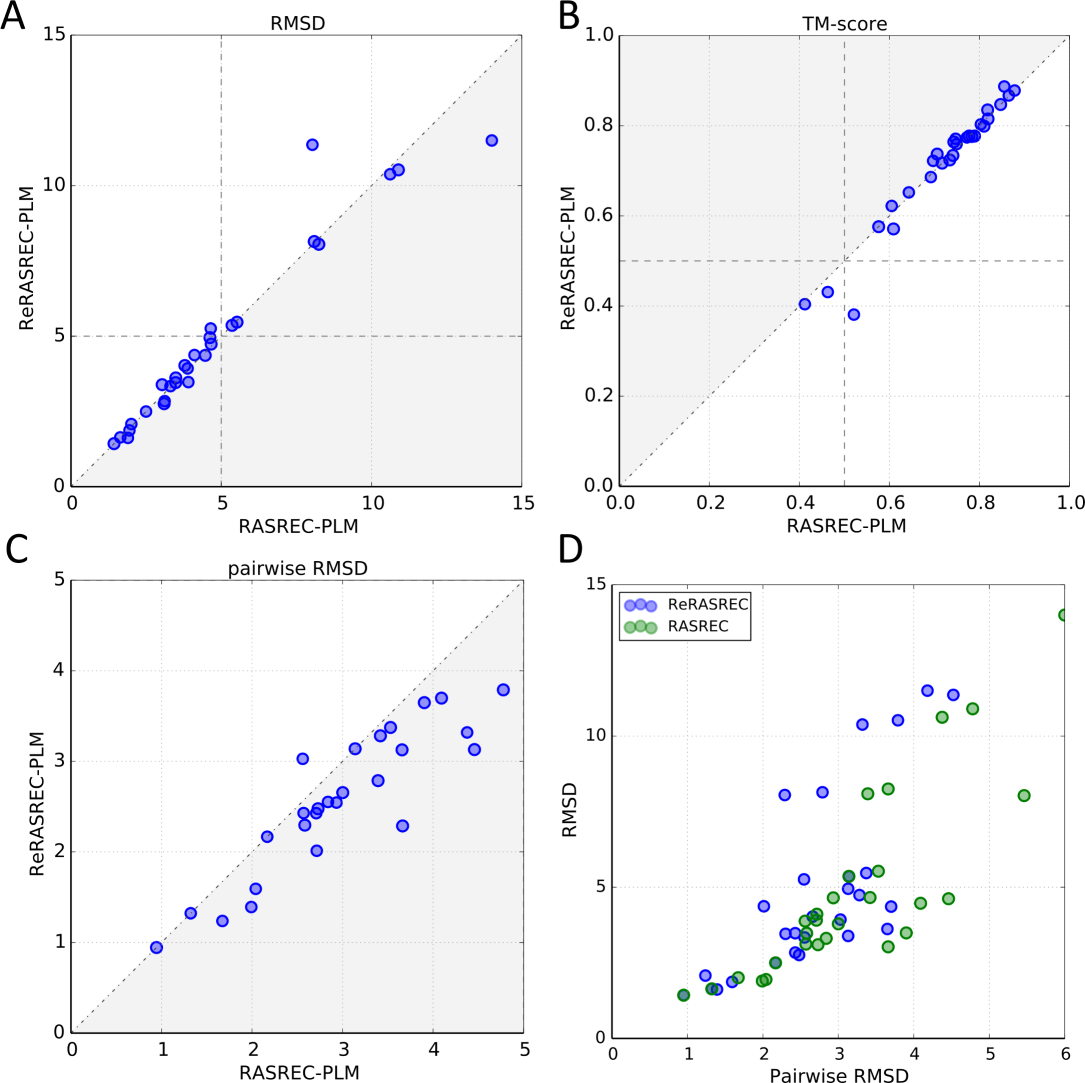
Comparison between initial RASREC results (RASREC-PLM) and refinement results (ReRASREC-PLM). A) RMSD and B) TM-scores of the 10 lowest-energy models of RASREC-PLM and ReRASREC-PLM C) Averaged pairwise RMSD of 10 lowest-energy models in ReRASREC-PLM and RASREC-PLM D) Average RMSD plotted against the average pairwise RMSD of the 10 lowest-energy models for both RASREC-PLM and ReRASREC-PLM.

Fig 6A and 6B show that the accuracy of the top ten scoring models after the refinement run did not significantly improve. However, Fig 6C indicates that the pairwise RMSD between all models in the ensemble of the 10 lowest-energy structures decreased by up to 1.4 Å after applying the refinement run, indicating better convergence. On average, the pairwise RMSD decreased by 0.5 Å. In addition, **Fig 6D** plots the average RMSD of the 10 lowest-energy models against their pairwise RMSD for both RASREC and ReRASREC. In both cases, a similar correlation between RMSD and pairwise RMSD can be observed. This shows that the refinement run does not lead to an artificial over-convergence but that the relation between both, as explored by RASREC individually, is kept.

This comparison shows that while the models have high accuracies after the initial RASREC run, the refinement run improves the overall prediction by increasing the precision and convergence of the final models.

### Convergence predicts accuracy

Fig 6D shows that there is a reasonable correlation between the pairwise RMSD and the overall performance of each target (pearson correlation coefficient of 0.83 and 0.73 for RASREC and ReRASREC respectively), meaning that low pairwise RMSD values correlate with low RMSD values and vice versa. The same trend can be observed when relating the backbone convergence (as defined previously) of a prediction to its performance, see Fig E in S1 Supporting Information: High backbone convergence corresponds to low RMSD values with a pearson correlation coefficient of -0.77. These strong correlations indicate that the accuracy of our final models can be predicted by their convergence. Highly converged structures (low pairwise RMSD) indicate an accurate prediction while a highly diverse ensemble suggests that the prediction is incorrect. This observation further reinforces our choice deeming predictions with a convergence lower than 50% as unsuccessful.

### Increase in prediction accuracy due to residue-residue contact information

**Fig 7:**
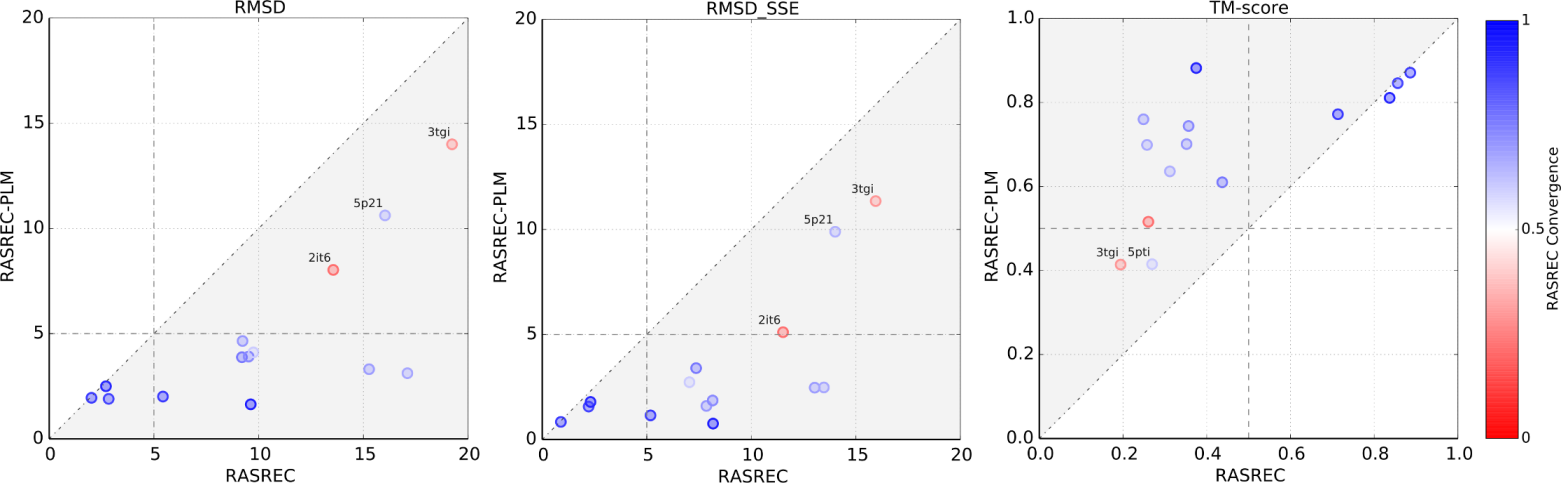
Comparison between RASREC runs without using contact information (RASREC) and RASREC runs using contacts predicted with the PLM approach (RASREC-PLM). For both methods, a single RASREC run without the optional refinement was carried out and the ensemble of the 10 lowest-energy models was considered as the final result. The color represents the fraction of converged residues in the 30 lowest energy models of RASREC-PLM.

To identify to what extent the RASREC protocol benefits from residue-residue contact information, we have compared RASREC runs without evolutionary information to RASREC runs including them in form of distance restraints for the 14 proteins of the EVFold benchmark set. For this test, we considered the results after a single RASREC run without the optional refinement step. As shown in Fig 7, without the use of evolutionary contact information, RASREC only predicted the fold of 3 out of 14 proteins correctly (TM Score > 0.5 or RMSD < 5Å) with an average TM-score of 0.41. However, if restraints derived from predicted residue-residue contacts were included, RASREC improved the coordinate accuracy for all targets of the benchmark set significantly, yielding an average TM-score over all 14 targets of 0.69. This shows that the additional data provided by the predicted residue-residue contacts enables RASREC to predict models in a near-native conformation, which would not be possible otherwise.

To investigate to what extend the RASREC protocol uses the available contact information, we compared the fraction of satisfied restraints (PPV), i.e. Cβ-Cβ distance ≤ 8 Å, in the top-scoring models of our protocol and the native structure (Fig F in S1 Supporting Information). On average, the fraction of satisfied restraints in the top-scoring models after the initial RASREC run (0.72) is very similar to the one of the native models (0.69). Overall, the RASREC models satisfy 88% of all restraints that are satisfied in the native structures, see Table D in S1 Supporting Information. RASREC furthermore correctly violates 63% of the incorrect distance restraints. The good correspondence between the PPVs on the native structure and the RASREC models, as well as the large fraction of satisfied “correct” restraints shows that RASREC is able to efficiently use the provided contact information. However, ignoring a larger amount of incorrect distance restraints might improve the prediction even further.

Comparing the PPVs, calculated for the restraints used by EVFold, on the top-ranked EVFold models and the native structures suggests that EVFold does not use the provided contact information as well as RASREC, see Fig F in S1 Supporting Information.

### Conclusions

In this study, we demonstrated that RASREC combined with evolutionary information is a powerful tool to predict the structures of globular proteins with high accuracy. Tested on a benchmark set of 28 globular proteins, we showed that our protocol is able to outperform latest state-of-the-art methods by predicting structures to higher accuracies for the majority of the benchmark set.

We further showed that the combination of improved sampling and high error tolerance of RASREC enables structure prediction in cases where the accuracy of predicted contacts is comparatively low, e.g. dropping below 50 percent. Robustness against erroneous distance restraints is of special interest for proteins for which only a limited amount of homologous sequences are known. The accuracy of residue-residue contact prediction is highly dependent on the number of available sequences in the multiple sequence alignment. For multiple sequence alignments with a small number of sequences, the accuracy is in general too low to significantly improve structure prediction using standard prediction protocols. We find that our protocol is able to more efficiently use the sparse information contained in contact predictions with low accuracy, due to the error robustness and iterative sampling strategy of the underlying RASREC algorithm. Our protocol should therefore be able to predict accurate models in cases where other currently published methods would most likely fail to predict the correct fold.

In addition, we have shown that integrating evolutionary information into the RASREC protocol is essential for accurate protein structure prediction for 9 out of 12 proteins in the EVFold benchmark set. Even adding contact predictions with accuracies as low as 45% can be sufficient to predict high resolution models that would not be possible using RASREC alone.

The optional refinement run improves the prediction by increasing the precision of the final models. Future work focusing on this step might further increase accuracy and convergence of the final models.

Overall, we have shown how evolutionary information can be efficiently used for predicting accurate protein structures. The rapid growth of sequence information and the current advances in statistical sequence analysis have made protein structure prediction using evolutionary information highly relevant. Finding a way to reliably and efficiently use the distance information contained in multiple sequence alignments will be a first step to fill the increasing gap between the large number of known protein sequences and the significantly smaller number of known protein structures.

## Supporting Information

S1 Supporting Information This Supporting Information file (PDF) contains supporting Figs A-F, Tables A-D, and Methods A-C. Fig A, Computational expense for the initial RASREC run. Fig B, Lowest-Energy ReRASREC-PLM Structures. Fig C, Comparison of ReRASREC-DI and EVFold-DI. Fig D, Comparison of top ranked ReRASREC models and lowest RMSD EVFold models, Fig E, Analysis of prediction performance and convergence. Fig F, Fraction of satisfied restraints in native structures and top-ranked models. Table A, TM-scores for EVFold, RASREC, PconsFold and FRAGFOLD. Table B, Accuracy of side-chain χ1 rotamers. Table C, Comparison between top ranked and lowest-RMSD structures. Table D, Restraint classification performance of RASREC. Method A, Contact Prediction and Restraint File Generation. Method B, Structure Prediciton with the RASREC protocol. Method C, Refinement with Rasrec.
(PDF)

S1 Text Protocol capture. This protocol capture describes the steps necessary to reproduce the results presented in the manuscript “Combining evolutionary information and an iterative sampling strategy for accurate protein structure prediction”. Exemplary input files and scripts to carry out the steps outlined in this protocol capture as well as exemplary output files are provided in S1 File. For simplification, we only describe structure prediction using our protocol for target 1wvn in this protocol capture. The supplementary materials are also included with Rosetta under the directory “Rosetta/demos/protocol_capture/2015/ rasrec_evolutionary_restraints”
(PDF)

S1 File Files for protocol capture. Input files for target 1wvn and scripts to carry out the steps outlined in the protocol capture in S1 Text as well as exemplary output files are provided in this attachement. The supplementary materials are also included with Rosetta under the directory “Rosetta/demos/protocol_capture/2015/ rasrec_evolutionary_restraints”
(ZIP)

S2 File Dataset. FASTA sequences and PLM contact predictions for all targets of the benchmark set.
(ZIP)
