# Supplementary material for: Combining Evolutionary Information and an Iterative Sampling Strategy for Accurate Protein Structure Prediction
Source: PLoS Comput Biol. 2015 Dec 29;11(12):e1004661. doi: 10.1371/journal.pcbi.1004661 (PMC4694711; doi:10.1371/journal.pcbi.1004661)
Supplement: S1 Supporting Information — Fig A, Computational expense for the initial RASREC run. Fig B, Lowest-Energy ReRASREC-PLM Structures. Fig C, Comparison of ReRASREC-DI and EVFold-DI. Fig D, Comparison of top ranked ReRASREC models and lowest RMSD EVFold models, Fig E, Analysis of prediction performance and convergence. Fig F, Fraction of satisfied restraints in native structures and top-ranked models. Table A, TM-scores for EVFold, RASREC, PconsFold and FRAGFOLD. Table B, Accuracy of side-chain χ1 rotamers. Table C, Comparison between top ranked and lowest-RMSD structures. Table D, Restraint classification performance of RASREC. Method A, Contact Prediction and Restraint File Generation. Method B, Structure Prediciton with the RASREC protocol. Method C, Refinement with Rasrec. (PDF) [file pcbi.1004661.s001.pdf]

SUPPLEMENT MATERIAL TO:

Combining evolutionary information and an iterative sampling strategy for accurate protein structure prediction

Tatjana Braun, Julia Koehler Leman, Oliver F. Lange

Table of Contents

Supporting Figures 2
Figure A: Computational expense for the initial RASREC run 2
Figure B: Lowest-Energy ReRASREC-PLM Structures 3
Figure C: Comparison of ReRASREC-DI and EVFold-DI 4
Figure D: Comparison of top-ranked ReRASREC structures and lowest-RMSD EVFold models 5
Figure E: Analysis of convergence and performance 6
Figure F: Fraction of satisfied restraints in the native and top-ranked models 7
Supporting Tables 8
Table A: TM-scores for EVFold, RASREC, PconsFold and FRAGFOLD 8
Table B: Accuracy of side-chain χ1 rotamers for EVFold-PLM and ReRASREC-PLM 9
Table C: Comparison between top ranked and lowest-RMSD structures 10
Table D: Restraint classification performance of RASREC 11
Supporting Methods 12
Method A: Contact Prediction and Restraint File Generation 12
Method B: Structure Prediciton with the RASREC protocol 12
Fragment Selection 12
Starting a RASREC run 12
Flag Files, Patches and Broker File 13
Method C: Refinement with Rasrec 16
Repick Restraints 16
Setup RASREC run 16
References 17

Supporting Figures

Figure A: Computational expense for the initial RASREC run

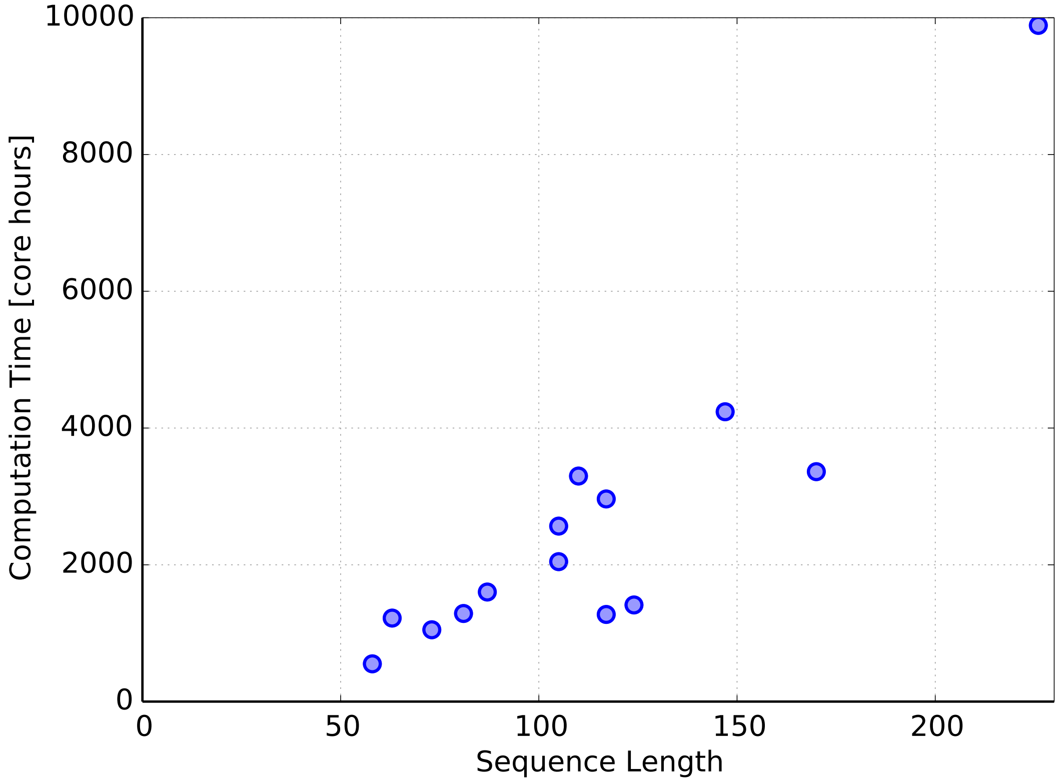

Figure A: Computational expense for the initial RASREC run. The computation time of the initial RASREC run for each of the targets of the EVFold benchmark set is plotted against the corresponding sequence length. The prediction has been carried out using a pool size of 500 on 2.6 GHz AMD Opteron processors. The computation time is dependent on several factors, including sequence length, fold complexity, and instructiveness of the restraints.

Figure B: Lowest-Energy ReRASREC-PLM Structures

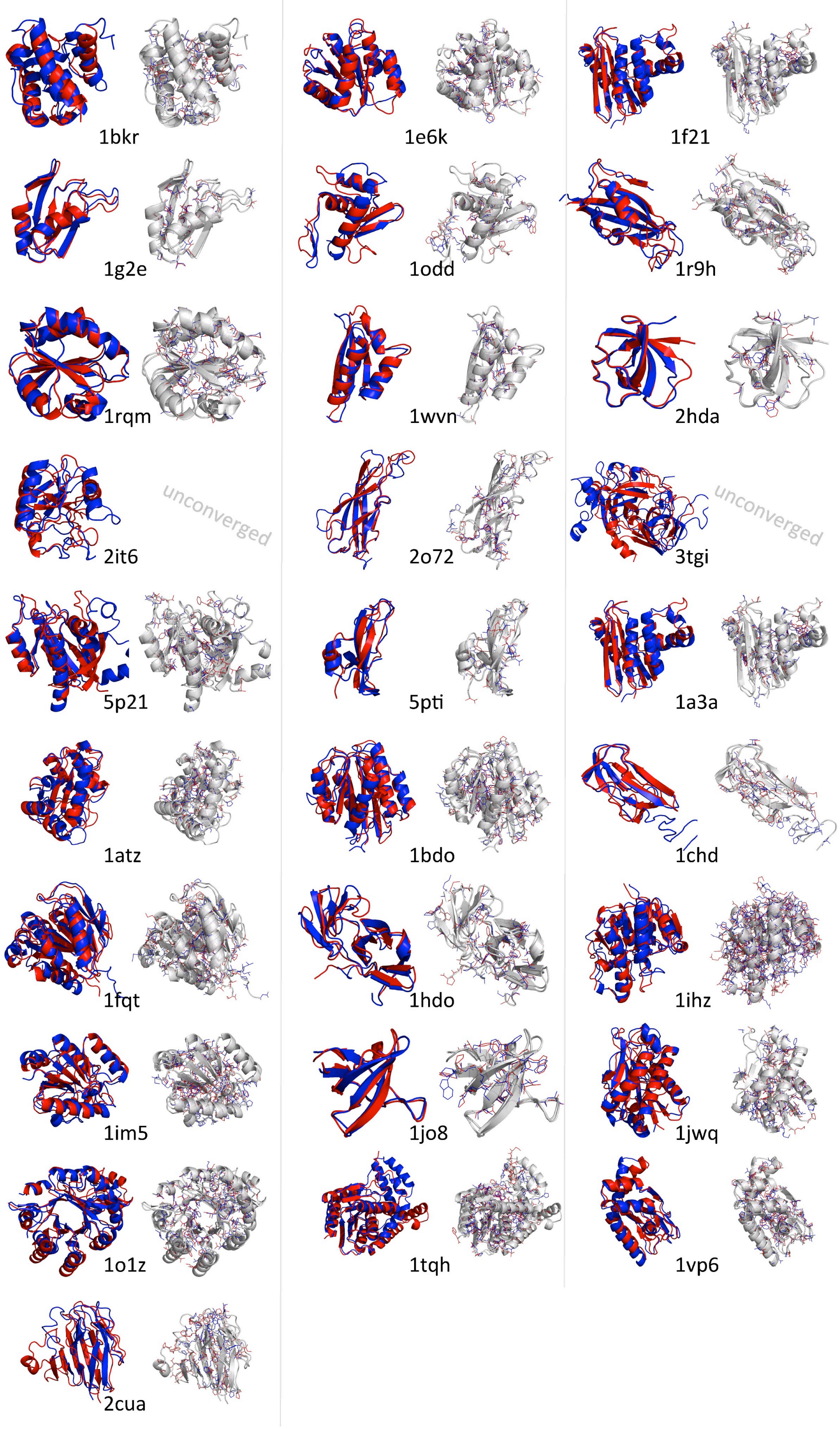

Figure B: Lowest-Energy ReRASREC-PLM Structures. The lowest-energy structures (red) are shown superimposed with the reference structure (blue). The same structures are shown with non-polar sidechains as lines in the right hand panel. If the RASREC calculation did not converge, the panel for sidechain details is omitted.

Figure C: Comparison of ReRASREC-DI and EVFold-DI

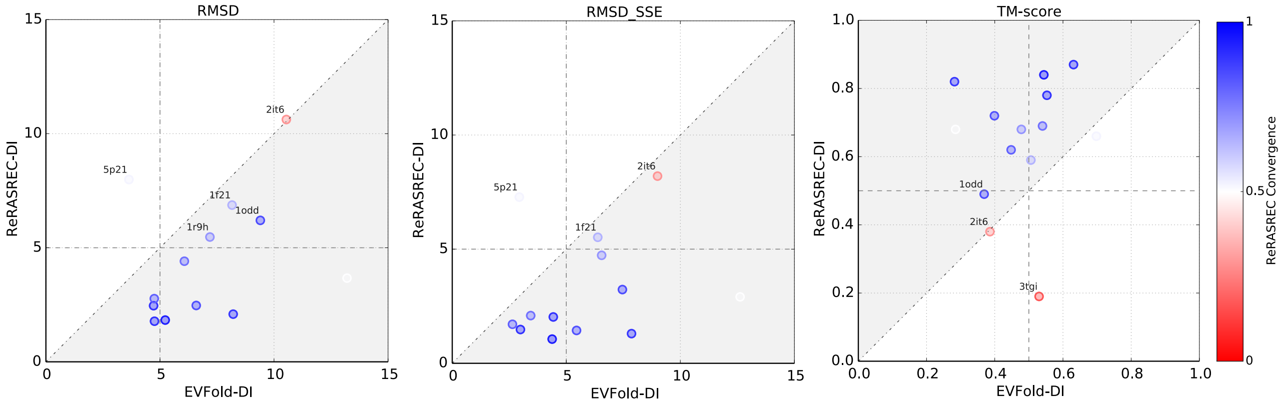

Figure C: Comparison of ReRASREC-DI and EVFold-DI. In case of ReRASREC-DI, the similarity measures are averaged over the 10 lowest-energy models, while for EVFold-DI the single top ranked model is evaluated. The color represents the fraction of converged residues in the 30 lowest-energy models of ReRASREC-DI. The gray areas indicate an improvement of ReRASREC-DI over EVFold-DI.

Figure D: Comparison of top-ranked ReRASREC structures and lowest-RMSD EVFold models

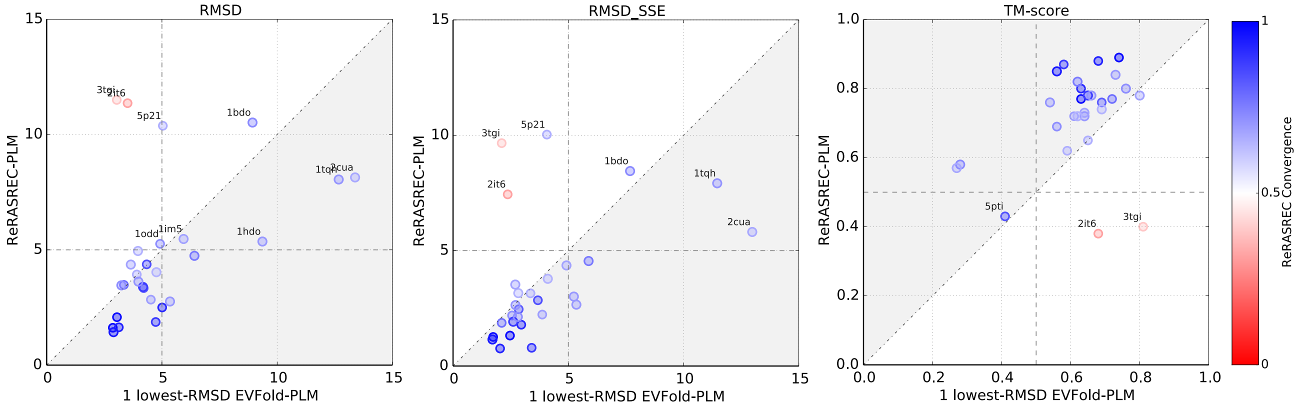

Figure D: Comparison of top-ranked ReRASREC models and lowest RMSD EVFold models. For each different metric, the single best performing EVFold structure (lowest RMSD) was selected among all 50 provided models and is compared to the average of this metric across the 10 lowest-energy models of a RASREC refinement run (ReRASREC-PLM)

Figure E: Analysis of convergence and performance

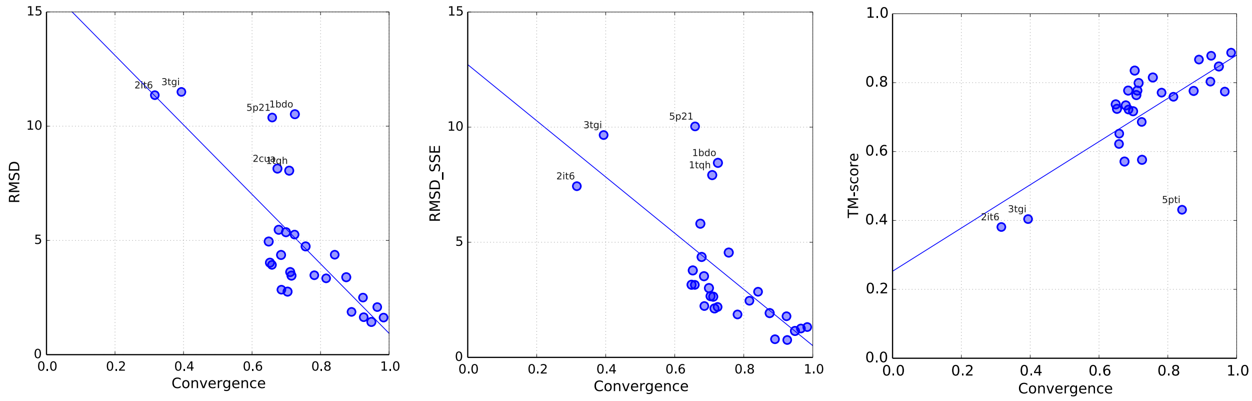

Figure E: Analysis of prediction performance and convergence. The different similarity measures (RMSD, RMSD_SSE, and TM-score) averaged over the 10 lowest energy models are compared to the fraction of converged residues in the 30 lowest energy models.

Figure F: Fraction of satisfied restraints in the native and top-ranked models

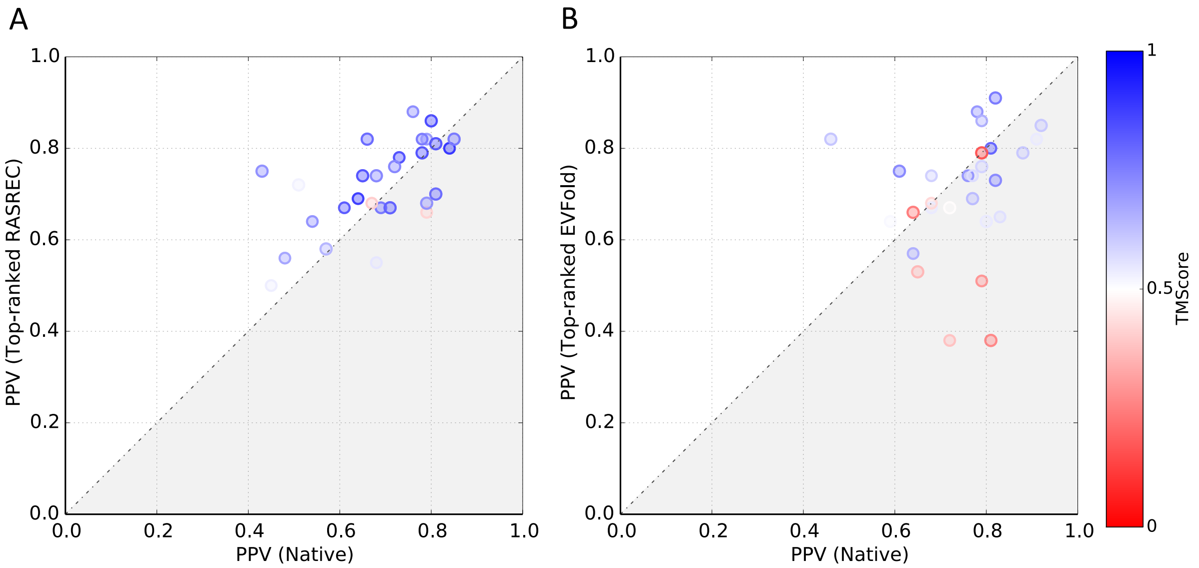

Figure F: Fraction of satisfied restraints in native structures and top-ranked models. PPVs were calculated for the restraint sets used for predicting the top-ranked models. Restraints were considered satisfied if the Cβ-Cβ distance is smaller or equal to 8Å. A) PPV values of restraints on top-ranked RASREC models (y-axis) and on the native structures (x-axis). For each target, L (=sequence length rounded down to the nearest multiple of 10) restraints were used. B) PPV values of Cβ-Cβ restraints on top-ranked EVFold models (y-axis) and on the native structures (x-axis). The restraints used for evaluation are the ones that have been used for generating the top-ranked EVFold model and the number therefore varies for each target between 10 and the sequence length.

Supporting Tables

Table A: TM-scores for EVFold, RASREC, PconsFold and FRAGFOLD

Benchmark set	Target	EVFold-PLM[*]	ReRASREC-PLM[†]	PconsFold (20k decoys)[‡]	FRAGFOLD[§]	Highest TM-score FAGFOLD[||]	Lowest-RMSD ReRASREC-PLM[¶]
EVFold	1bkr	0.30	0.62	**0.74**	N/A	<0.76	<0.69
	1e6k	0.71	**0.89**	0.82	N/A	<0.85	<0.91
	1f21	0.70	**0.76**	0.61	N/A	<0.58	<0.82
	1g2e	0.56	**0.88**	0.80	N/A	<0.80	<0.91
	1odd	0.51	**0.69**	0.59	N/A	<0.62	<0.76
	1r9h	0.57	**0.72**	0.65	N/A	<0.91	<0.81
	1rqm	0.54	0.80	**0.83**	N/A	<0.80	<0.85
	1wvn	0.54	**0.87**	0.60	N/A	<0.70	<0.89
	2hda	0.42	**0.77**	0.57	N/A	<0.85	<0.87
	2it6	**0.66**	0.38	0.54	N/A	<0.45	<0.42
	2o72	0.65	**0.77**	0.53	N/A	-	<0.80
	3tgi	**0.80**	0.40	0.54	N/A	<0.51	<0.42
	5p21	0.59	0.65	**0.67**	N/A	<0.88	<0.66
	5pti	0.38	0.43	**0.57**	N/A	<0.57	<0.55
	Mean	0.57	**0.69**	0.65	N/A	<0.71	<0.74
Pconsfold	1a3a	0.61	**0.74**	N/A	0.56	N/A	<0.77
	1atz	0.73	**0.84**	N/A	0.64	N/A	<0.88
	1bdo	0.25	**0.58**	N/A	0.44	N/A	<0.59
	1chd	0.76	**0.78**	N/A	0.65	N/A	<0.79
	1fqt	0.61	**0.78**	N/A	0.7	N/A	<0.88
	1hdo	0.61	**0.72**	N/A	0.56	N/A	<0.73
	1ihz	0.63	**0.78**	N/A	0.62	N/A	<0.81
	1im5	0.59	**0.73**	N/A	0.56	N/A	<0.78
	1jo8	0.49	**0.85**	N/A	0.84	N/A	<0.9
	1jwq	0.73	**0.80**	N/A	0.31	N/A	<0.84
	1o1z	0.17	**0.82**	N/A	0.61	N/A	<0.85
	1tqh	0.54	**0.76**	N/A	0.5	N/A	<0.77
	1vp6	0.36	**0.72**	N/A	0.52	N/A	<0.85
	2cua	0.27	**0.57**	N/A	0.56	N/A	<0.61
	Mean	0.52	**0.75**	N/A	0.58	N/A	<0.79

Table A: TM-scores for EVFold, RASREC, PconsFold and FRAGFOLD. Column 1-4 show the top ranked results for methods EVFold-PLM, ReRASREC-PLM, PconsFold and FRAGFOLD. Column 5 furthermore shows the highest TM-scores obtained with FRAGFOLD and Column 6 the average TM-score of the ensemble of 10 lowest-RMSD models obtained with ReRASREC-PLM. The numbers in these two columns are preceeded by '<' as they do not reflect the 'real' (best ranked) results, but the ones closest to the native and are therefore not comparable to the rest.
The highest TM-score for each target amongst the top-ranked results (Column 1-4) is highlighted.
[*] Single top ranked results using the webserver of EVFold with PLM-restraints
[†] Ensemble of 10 lowest-energy models of ReRASREC using PLM-restraints
[‡] Top ranked models obtained with PconsFold. Restraints have been generated with PconsC. Values are taken from (Michel et al., 2014).
[§] Highest TM-score amongst top 5 ranked models using FRAGFOLD (using all contacts). Restraints have been generated with PSICOV. Values are taken from (Kosciolek & Jones, 2014).
[||] Highest TM-scores obtained with FRAGFOLD. Restraints have been generated with PSICOV. Values are taken from (Kosciolek & Jones, 2014).
[¶] Ensemble of 10 lowest-RMSD structures obtained with ReRASREC-PLM.

Table B: Accuracy of side-chain χ1 rotamers for EVFold-PLM and ReRASREC-PLM

Benchmark set	Target	buried*	Recovered χ1 Rotamers					
			ReRASREC-PLM		EVFold-PLM		EVFold-PLM (relaxed)	
			Recovered χ1 †	Fraction of rec. χ1 ‡	Recovered χ1 †	Fraction of rec. χ1 ‡	Recovered χ1 †	Fraction of rec. χ1 ‡
**EVFold benchmark set**	1bkr	42	28	0.67	14	0.33	16	0.38
	1e6k	49	39	0.80	27	0.55	25	0.51
	1f21	53	38	0.72	32	0.60	37	0.70
	1g2e	25	19	0.76	11	0.44	13	0.52
	1odd	27	19	0.70	13	0.48	13	0.48
	1r9h	36	28	0.78	12	0.33	16	0.44
	1rqm	42	25	0.60	22	0.52	21	0.50
	1wvn	19	14	0.74	6	0.32	7	0.37
	2hda	16	9	0.56	5	0.31	11	0.69
	2it6	48	24	0.50	21	0.44	26	0.54
	2o72	27	20	0.74	11	0.41	10	0.37
	3tgi	101	52	0.51	44	0.44	49	0.49
	5p21	71	45	0.63	31	0.44	38	0.54
	5pti	14	5	0.36	8	0.57	8	0.57
**Pconsfold benchmark set**	1a3a	56	37	0.66	23	0.41	27	0.48
	1atz	72	47	0.65	29	0.40	34	0.47
	1bdo	25	15	0.60	8	0.32	11	0.44
	1chd	74	50	0.68	32	0.43	37	0.50
	1fqt	44	32	0.73	16	0.36	21	0.48
	1hdo	84	46	0.55	30	0.36	38	0.45
	1ihz	51	35	0.69	26	0.51	25	0.49
	1im5	68	40	0.59	27	0.40	32	0.47
	1jo8	15	11	0.73	4	0.27	6	0.40
	1jwq	76	47	0.62	30	0.39	40	0.53
	1o1z	99	70	0.71	34	0.34	58	0.59
	1tqh	106	79	0.75	43	0.41	50	0.47
	1vp6	50	39	0.78	25	0.50	27	0.54
	2cua	46	25	0.54	15	0.33	20	0.43
**Average**	N/A	N/A	33.5	0.65	21.4	0.41	25.6	0.49

Table B: Accuracy of side-chain χ1 rotamers. Buried side chains in single top-ranked models are selected and their adopted rotamer assignments are compared to those in the reference crystal structure. In case of EVFold, the analysis has been carried out for the models as generated by CNS (EVFold-PLM) and after relaxing them with fixed backbone atoms within the Rosettal full-atom energy (EVFold-PLM (relaxed)). Glycine and Alanine are excluded from this analysis.

* Side chains that are buried in the reference structure (SASA < 40Å)

† Side chains that are buried in the reference structure and have the same χ1 rotamer assignment in the top-ranked models as in the reference structure

‡ Fraction of buried side chains with the the same χ1 rotamer assignment in the top-ranked models and the reference structure

Table C: Comparison between top ranked and lowest-RMSD structures

		RMSD								TM-score							
		ReRASREC-PLM				EVFold-PLM				ReRASREC-PLM				EVFold-PLM			
Bench-mark set	Target	top 10*	best 10†	top 1‡	best 1§	top 10*	best 10†	top 1‡	best 1§	top 10*	best 10†	top 1‡	best 1§	top 10*	best 10†	top 1‡	best 1§
EVFold bench-mark set	1bkr	3.93	3.06	4.11	2.93	9.52	4.77	13.79	3.91	0.62	0.69	0.60	0.72	0.41	0.54	0.30	0.59
	1e6k	1.62	1.37	1.57	1.27	3.44	3.10	3.34	2.87	0.89	0.92	0.90	0.93	0.70	0.72	0.71	0.74
	1f21	3.34	2.63	2.87	2.56	4.84	4.40	4.21	4.21	0.76	0.82	0.81	0.83	0.65	0.67	0.70	0.69
	1g2e	1.64	1.09	1.48	0.82	3.83	3.39	4.23	3.13	0.88	0.92	0.89	0.94	0.59	0.65	0.56	0.68
	1odd	5.26	3.27	5.46	2.93	6.15	5.28	6.14	4.92	0.69	0.76	0.67	0.79	0.50	0.53	0.51	0.56
	1r9h	2.84	2.05	2.51	1.85	5.93	4.86	4.87	4.52	0.72	0.82	0.76	0.84	0.52	0.56	0.57	0.61
	1rqm	2.50	1.77	2.26	1.63	7.23	5.73	5.91	5.01	0.80	0.86	0.82	0.86	0.52	0.57	0.54	0.63
	1wvn	1.87	1.35	2.01	1.29	5.83	5.26	5.87	4.73	0.87	0.89	0.85	0.90	0.51	0.52	0.54	0.58
	2hda	2.08	1.36	2.03	1.30	4.64	3.60	4.91	3.05	0.77	0.87	0.84	0.89	0.49	0.53	0.42	0.63
	2it6*	11.36	9.54	11.74	9.17	4.31	3.72	3.94	3.51	0.38	0.42	0.37	0.45	0.60	0.66	0.66	0.68
	2o72	3.48	2.84	2.86	2.62	4.13	3.95	4.14	3.35	0.77	0.80	0.82	0.82	0.66	0.67	0.65	0.72
	3tgi*	11.50	10.04	11.17	9.75	3.53	3.19	3.12	3.04	0.40	0.42	0.40	0.43	0.77	0.80	0.80	0.81
	5p21	10.38	8.78	10.70	7.88	7.04	5.50	6.58	5.04	0.65	0.66	0.65	0.67	0.57	0.61	0.59	0.65
	5pti	4.37	3.27	4.69	2.92	8.65	4.93	5.82	4.34	0.43	0.55	0.43	0.60	0.27	0.37	0.38	0.41
Pcons-Fold bench-mark set	1a3a	4.95	3.47	3.38	2.62	5.30	4.37	5.28	3.96	0.74	0.77	0.78	0.83	0.64	0.68	0.61	0.69
	1atz	2.76	2.21	2.44	2.15	9.93	6.52	5.35	5.21	0.84	0.89	0.87	0.89	0.61	0.71	0.73	0.73
	1bdo	10.52	8.17	11.73	7.54	17.67	10.66	11.39	8.93	0.58	0.59	0.56	0.58	0.25	0.25	0.25	0.28
	1chd	4.36	3.98	4.31	3.78	4.22	3.97	4.10	3.65	0.78	0.79	0.78	0.81	0.77	0.79	0.76	0.80
	1fqt	3.39	1.70	2.24	1.53	4.88	4.76	5.36	4.18	0.78	0.88	0.82	0.89	0.61	0.61	0.61	0.65
	1hdo	5.36	4.79	5.57	4.74	10.53	9.49	9.46	9.36	0.72	0.73	0.71	0.73	0.58	0.61	0.61	0.64
	1ihz	3.62	2.68	3.90	2.46	5.00	4.49	4.46	3.98	0.78	0.81	0.76	0.81	0.61	0.63	0.63	0.66
	1im5	5.47	3.93	5.69	3.59	8.03	6.54	7.30	5.94	0.73	0.78	0.70	0.81	0.58	0.62	0.59	0.64
	1jo8	1.43	0.95	1.54	0.84	4.55	3.19	3.56	2.90	0.85	0.90	0.85	0.91	0.47	0.54	0.49	0.56
	1jwq	3.46	2.86	3.50	2.71	3.54	3.41	3.57	3.24	0.80	0.84	0.79	0.85	0.73	0.74	0.73	0.76
	1o1z	4.74	4.24	4.95	4.21	15.25	8.69	25.41	6.41	0.82	0.85	0.80	0.84	0.39	0.55	0.17	0.62
	1tqh	8.05	5.20	7.98	4.33	15.82	13.44	12.67	12.67	0.76	0.78	0.78	0.79	0.48	0.53	0.54	0.54
	1vp6	4.03	2.03	2.75	1.93	12.58	9.04	10.95	4.76	0.72	0.85	0.82	0.86	0.35	0.44	0.36	0.62
	2cua	8.14	7.10	8.32	6.99	19.94	18.27	19.22	14.18	0.57	0.61	0.57	0.61	0.29	0.30	0.27	0.27
**Average**		**4.87**	**3.78**	**4.78**	**3.51**	**7.72**	**6.02**	**7.32**	**5.18**	**0.72**	**0.77**	**0.73**	**0.78**	**0.54**	**0.59**	**0.55**	**0.62**

Table C: Comparison between top ranked and lowest-RMSD structures.

* ensemble of 10 lowest-energy (ReRASREC-PLM)/ best ranked (EVFold-PLM) models

† ensemble of 10 lowest-RMSD models

‡ single lowest-energy (ReRASREC-PLM)/ best ranked (EVFold-PLM) models

§ single lowest-RMSD models

Table D: Restraint classification performance of RASREC

Benchmark Set	Target	#Restraints	TP	FP	TN	FN	TPR= TP/(TP+FN)	TNR= TN/(TN+FP)	PPV= TP/(TP+FP)	NPV= TN/(TN+FN)	ACC= (TP+TN)/(P+N)
EVFold Benchmark Set	**1bkr**	110	40	15	45	10	0.80	0.75	0.73	0.82	0.77
	**1e6k**	120	81	12	20	7	0.92	0.63	0.87	0.74	0.84
	**1f21**	140	79	15	28	18	0.81	0.65	0.84	0.61	0.76
	**1g2e**	80	63	1	12	4	0.94	0.92	0.98	0.75	0.94
	**1odd**	80	38	13	24	5	0.88	0.65	0.75	0.83	0.78
	**1r9h**	100	70	12	9	9	0.89	0.43	0.85	0.50	0.79
	**1rqm**	100	55	12	27	6	0.90	0.69	0.82	0.82	0.82
	**1wvn**	70	45	3	22	0	1.00	0.88	0.94	1.00	0.96
	**2hda**	50	36	5	6	3	0.92	0.55	0.88	0.67	0.84
	**2it6**	110	50	11	24	25	0.67	0.69	0.82	0.49	0.67
	**2o72**	110	80	17	9	4	0.95	0.35	0.82	0.69	0.81
	**3tgi**	220	125	21	25	49	0.72	0.54	0.86	0.34	0.68
	**5p21**	170	73	23	66	8	0.90	0.74	0.76	0.89	0.82
	**5pti**	60	30	11	9	10	0.75	0.45	0.73	0.47	0.65
Pconsfold Benchmark Set	**1a3a**	140	87	8	22	23	0.79	0.73	0.92	0.49	0.78
	**1atz**	180	134	12	22	12	0.92	0.65	0.92	0.65	0.87
	**1bdo**	80	38	20	19	3	0.93	0.49	0.66	0.86	0.71
	**1chd**	200	131	9	30	30	0.81	0.77	0.94	0.50	0.81
	**1fqt**	110	84	6	10	10	0.89	0.63	0.93	0.50	0.85
	**1hdo**	200	78	72	41	9	0.90	0.36	0.52	0.82	0.60
	**1ihz**	140	99	11	20	10	0.91	0.65	0.90	0.67	0.85
	**1im5**	180	116	20	30	14	0.89	0.60	0.85	0.68	0.81
	**1jo8**	50	40	3	7	0	1.00	0.70	0.93	1.00	0.94
	**1jwq**	170	100	26	33	11	0.90	0.56	0.79	0.75	0.78
	**1o1z**	230	138	16	51	25	0.85	0.76	0.90	0.67	0.82
	**1tqh**	240	149	29	48	14	0.91	0.62	0.84	0.77	0.82
	**1vp6**	130	85	21	23	1	0.99	0.52	0.80	0.96	0.83
	**2cua**	130	63	12	44	11	0.85	0.79	0.84	0.80	0.82
**Average**		-	-	-	-	-	0.88	0.63	0.84	0.70	0.80

Table D: Restraint classification performance of RASREC. Evaluation is carried out for the single top-ranked RASREC models. Restraint sets are the ones used for model generation. A restraint is defined as correct (P) if the Cβ-Cβ distance in the native structure is ≤ 8Å, otherwise as incorrect (N).

TP: Restraints satisfied in both model and native structure

FP: Restraints satisfied in model, but not in the native structure

TN: Restraints neither satisfied in model nor native structure

FN: Restraints satisfied in native structure, but not in model

Supporting Methods

Detailed instructions about how to recreate and analyze the results presented in the manuscript can be found in the protocol capture (provided as File S2 and in the current Rosetta release). The protocol capture contains all necessary flag files, command lines and scripts.

Method A: Contact Prediction and Restraint File Generation

The contact predictions used in this manuscript have been generated with the EVFold webserver (Marks et al., 2011) (available at http://evfold.org/evfold-web/newprediction.do) using standard parameters. The results can be downloaded in form of a compressed folder, which is subdivided into several subdirectories. The all-by-all residue pairing scores are stored in `{jobname}_{scoringmethod}.txt` in the subfolder `ev_couplings`. In case of PLM as scoring method, the file is named `{jobname}_PLM.txt`.

From this file, the L top-ranked residue pairing scores having a minimum distance of 5 residues are extracted and translated into Rosetta specific distance restraints with a sigmoidal potential.

Exemplary excerpt from the generated distance restraint file:

```
AtomPair   CA   97   CB  117  SIGMOID 8.00 1.00 #ContactMap: 0.82
AtomPair   CB   18   CB   47  SIGMOID 8.00 1.00 #ContactMap: 0.78
AtomPair   CB   89   CB  113  SIGMOID 8.00 1.00 #ContactMap: 0.77
```

Method B: Structure Prediciton with the RASREC protocol

Fragment Selection

We have run the fragment picker for all targets with the following command:
`make_fragments.pl -nohoms`

The flag `-nohoms` leads to exclusion of fragments from homologous proteins. This flag should be omitted when not used for benchmarking.

Starting a RASREC run

The structures are generated with the RASREC protocol (Lange & Baker, 2012) of the molecular modeling suite Rosetta (using commit #aa72710 from March 2014) with a pool size of 500. A detailed list of all commands used for a RASREC calculation can be found below.

```
mpiexec -np <CORES> minirosetta.mpi.linuxgccrelease -out:file:silent decoys.out
@flags_denovo @flags_rasrec @flags_iterative -run:archive -out:nstruct <CORES-
3>
```

Flag Files, Patches and Broker File

Command-line flags and patches are separated into a number of different files. All parameters used to generate the data in our manuscript are listed below.

For executing RASREC, bold elements need to be replaced with actual input files.

flags_denovo

```
-run:protocol broker

#fragment files
-frag3 <frags.3mers>
-frag9 <frags.9mers>
#input fasta sequence
-in:file:fasta <sequence.fasta>

-out:file:silent_print_all_score_headers
-increase_cycles 2.000000

#jumping
-templates::topology_rank_cutoff 0.8
-jumps:ramp_chainbreaks
-jumps:sep_switch_accelerate 0.8
-abinitio:skip_convergence_check
-jumps:overlap_chainbreak

#energy fixes
-rsd_wt_helix 0.5
-rsd_wt_loop 0.5
-rg_reweight 0.5

#for loop closing
-overwrite_filter_scorefxn score3
-detect_disulf false

#loop-closing filter in SlidingWindow
-fast_loops:overwrite_filter_scorefxn score3

-abrelax:fail_unclosed

#specify logfile output level
-unmute memory_usage
-out:levels core.chemical:error
-out:levels core.io.pdb:error
-out:levels protocols.jobdist:error

#load flags in flags_nmr_patches
@flags_nmr_patches
```

flags_fullatom

```
-relax:fast
-relax:ramady
-abinitio:close_loops
-loops:idealize_before_loop_close
-loops:idealize_after_loop_close
-abinitio::clear_pose_cache
-short_frag_cycles 1
-scored_frag_cycles 1
```

```
-non_ideal_loop_closing
-alternative_closure_protocol
-fast_loops:window_accept_ratio .01
-fast_loops:nr_scored_sampling_passes 4
-fast_loops:min_breakout_good_loops 5
-fast_loops:min_breakout_fast_loops 80
-fast_loops:min_fast_loops 3
-fast_loops:min_good_loops 0
-fast_loops:nr_scored_fragments 20
-fast_loops:vdw_delta 0.5
-fast_loops:give_up 1000
```

<u>flags_iterative</u>

```
-iterative:enumerate:skip_half
#RASREC pool size
-iterative:pool_size 500
#Acceptance ratio for different RASREC stages
-iterative:accept_ratio 0.1 0.1 0.1  0.1 0.1 0.1 0.1 0.1
-jumps::max_strand_gap_allowed 10
-jumps:contact_score 0.2
-iterative:rmsf_nstruct 50
#Output levels for log file
-out:level 400
-out:levels all:warning
-out:levels protocols.jd2.MPIArchiveJobDistributor:info
-out:levels protocols.jd2.Archive:debug
-out:levels protocols.iterative:info
-out:levels core.util.prof:info

#obsolete
-iterative:evaluate_only_on_slaves

#scoring functions for fullatom and centroid stages
-iterative:fa_score talaris2013
-iterative:cen_score score3

#Stages:
# (1)  SS-RANDOM
# (2)  MIX
# (3)  BETA-TOP
# (4)  RESAM
# (5)  NOE-BETA-TOP
# (6)  NOE-RESAM
# (7)  CEN2FULL
# (8)  FULL-REFINE

-iterative:max_nstruct 0 0 0 0 -1 -1 0 0
-iterative:min_diversity 0 0 0 2.0 3.0 2.0 2.0 1.5
-iterative:fullatom

-iterative:safety_hatch_scorecut 0.1
-iterative::super_quick_relax_patch super_quick_relax.patch

#this is the relative weight the noesy-cst will have for filtering
#the relative weight provided in the following is multiplied with the overall
#weight for atom_pair_constraint in the patches
#given by -iterative:cen_score_patch and -iterative:fa_score_patch
-iterative:cenpool_noesy_cst_weight 1
-iterative:fapool_noesy_cst_weight 1

#exit as soon as queue is drained
```

```
-jd2:mpi_nowait_for_remaining_jobs
-jd2:mpi_timeout_factor 0

-iterative:flags_fullatom flags_fullatom

#important to obtain intermediate structures for proto-fold resampling (aka
stage2 resampling)
-abinitio:debug
-abinitio:debug_structures

-archive:completion_notify_frequency 125
```

flags_rasrec

```
#File containing information about distance restraints
-broker:setup setup_init.tpb

#Only needed for evaluation purposes (in case native structure is known)
-in:file:native <native.pdb>
-evaluation:rmsd NATIVE _full <native.rigid>
```

flags_nmr_patches

```
#patches used for abinitio stages
-abinitio::stage2_patch nmr_patch
-abinitio::stage3a_patch nmr_patch
-abinitio::stage3b_patch nmr_patch
-abinitio::stage4_patch nmr_patch

#for fullatom-relax
-score::patch nmr_relax_patch

# for loop closing
-fast_loops:patch_filter_scorefxn nmr_patch
-patch_filter_scorefxn nmr_patch

-iterative:fa_score_patch  nmr_pool_patch
-iterative:cen_score_patch nmr_pool_patch
```

nmr_patch

```
atom_pair_constraint = 5.0
rdc    = 5.0
```

nmr_pool_patch

```
chainbreak = 1
linear_chainbreak = 1.33
overlap_chainbreak = 1
atom_pair_constraint = 10
rdc    = 10
```

nmr_relax_patch

```
atom_pair_constraint = 0.1
rdc    = 0.1
```

setup_init.tpb

```
CLAIMER ConstraintClaimer
file <restraints.cst>
FULLATOM
```

```
CENTROID
SKIP_REDUNDANT 0
FILTER_WEIGHT  1.00
FILTER_NAME restraints_SIGMOID
END_CLAIMER
```

Method C: Refinement with Rasrec

If the convergence of the initial RASREC run was not sufficiently high (fraction of converged residues < 90%), a second RASREC run was carried out. This run reuses restraints from both predicted contact map and the previous results.

Repick Restraints

The refinement run uses restraints from both predicted contact map and the previous results. For this purpose, two different restraint files have been generated:

`target_converged_distances.cst`
This restraint file contains all short-range (<=8 Å) distances with a standard deviation < 1 Å in the 30 lowest-energy RASREC models. These converged distances will be enforced during ReRASREC using a strict bounded potential. To reflect the average distance *d* in the converged region, the lower bound is set to (*d*-1) and the upper bound to (*d*+1).

`target_filtered_contactmaps.cst`
This file contains additional residue-residue pairings from the predicted contact map that affect unconverged regions (residue-residue distance, SD > 1Å in 30 low-energy structures) and do not totally disagree (i.e. are short range with an average distance d $\leq$ 8Å) with the preliminary structures. The restraints are scored with a wide bounded potential with lower bound and upper bound set to 1.5Å and 8Å respectively. Random pairs of these restraints are combined into ambiguous restraints (see below).

Setup RASREC run

The flags and patches used for the refinement RASREC run are identical to the ones listed in Method B: Structure Prediciton with the RASREC protocol. The two RASREC runs only differ in the restraints used for structural guiding. The restraint files are added to a RASREC run in the broker file `setup_init.tpb`.

Both restraint files that have been generated above will be added to the broker file for the refinement run as follows

```
CLAIMER ConstraintClaimer
file target_converged_distances.cst
FULLATOM
CENTROID
SKIP_REDUNDANT 0
FILTER_WEIGHT  1.00
FILTER_NAME converged_distances
END_CLAIMERls
```

```
CLAIMER ConstraintClaimer
file target_filtered_contactmaps.cst
FULLATOM
CENTROID
COMBINE_RATIO 2   #make the restriants ambiguous
SKIP_REDUNDANT 0
FILTER_WEIGHT  1.00
FILTER_NAME filtered_contactmaps
END_CLAIMER
```

Please note, that for the filtered_contactmap.cst restraints the following line is added:
`COMBINE_RATIO 2`
This line transforms the restraints to ambiguous ones.

References

1. Michel M, Hayat S, Skwark MJ, Sander C, Marks DS, Elofsson A (2014) PconsFold: improved contact predictions improve protein models. Bioinformatics 30: i482-8.

2. Kosciolek T, Jones DT (2014) De novo structure prediction of globular proteins aided by sequence variation-derived contacts. PLoS One 9: e92197.

3. Marks DS, Colwell LJ, Sheridan R, Hopf TA, Pagnani A, Zecchina R, et al. (2011) Protein 3D structure computed from evolutionary sequence variation. PLoS One 6: e28766.

4. Lange OF, Baker D (2012) Resolution-adapted recombination of structural features significantly improves sampling in restraint-guided structure calculation. Proteins 80: 884-95.
