# Supplementary material for: Combining Evolutionary Information and an Iterative Sampling Strategy for Accurate Protein Structure Prediction
Source: PLoS Comput Biol. 2015 Dec 29;11(12):e1004661. doi: 10.1371/journal.pcbi.1004661 (PMC4694711; doi:10.1371/journal.pcbi.1004661)
Supplement: S1 Text — This protocol capture describes the steps necessary to reproduce the results presented in the manuscript “Combining evolutionary information and an iterative sampling strategy for accurate protein structure prediction”. Exemplary input files and scripts to carry out the steps outlined in this protocol capture as well as exemplary output files are provided in S1 File. For simplification, we only describe structure prediction using our protocol for target 1wvn in this protocol capture. The supplementary materials are also included with Rosetta under the directory “Rosetta/demos/protocol_capture/2015/ rasrec_evolutionary_restraints” (PDF) [file pcbi.1004661.s002.pdf]

PROTOCOL CAPTURE – Combining Evolutionary Information and an Iterative Sampling Strategy for Accurate Protein Structure Prediction

General Description

This protocol capture has been written by Tatjana Braun in January 2015 and demonstrates on an exemplary target how the results presented in the RosettaCon collection paper "Combining evolutionary information and an iterative sampling strategy for accurate protein structure prediction" by Tatjana Braun, Julia Koehler Leman and Oliver Lange (2015) have been generated.
The protocol capture has been tested with a Rosetta version from October 2014.

This protocol capture (including all scripts necessary to run RASREC in combination with evolutionary restraints) is available in all Rosetta releases published after March 2015 in the following directory:
`Rosetta/demos/protocol_capture/2015/rasrec_evolutionary_restraints`

Directory structure

The following files and folders are provided in this protocol capture

scripts/	python scripts for generating distance restraints, analyzing the final results and setting up a RASREC run
rosetta_flags/	all necessary flagfiles for setting up the initial and refinement RASREC run
inputs/	All necessary input files, including EVFold prediction, FASTA file, fragment files and native structure
outputs	Exemplary outputs for all steps carried out in this tutorial

Detailed Instructions

The following section is written in style of a tutorial and reflects exactly what has been done for each target in the manuscript. This tutorial will predict the structure of 1wvn with all necessary input files provided. All commands listed in this tutorial can directly be executed from the `protocol_capture` folder if the path of this tutorial is stored in the following variable:

```
PROTOCOL=~/path/to/tutorial
cd $PROTOCOL
```

1) Contact Prediction and Restraint File Generation

The contact predictions used in the manuscript have been generated with the EVFold webserver (available at http://evfold.org/evfold-web/newprediction.do) using standard parameters. The results can be downloaded in form of a compressed folder, which is subdivided into several subdirectories. The all-by-all residue pairing scores are stored in `{jobname}_{scoringmethod}.txt` in the `ev_couplings` folder. In case of the standard scoring method (PLM), the file is named `{jobname}_PLM.txt`.

An exemplary prediction for 1wvn is provided in inputs/ev_couplings/1wvn_PLM.txt.

From this score file, the l top-ranked residue pairing scores having a minimum distance of 5 residues are extracted and translated into Rosetta specific distance restraints.

This can be done with the following two steps:

1.1) Generate lxl contact map

```
$PROTOCOL/scripts/create_evfold_contactmap.py \\
      -i inputs/ev_couplings/1wvn_PLM.txt \\
      -f inputs/1wvn.fasta \\
      -o 1wvn_PLM.cmp
```

This command generates a file called 1wvn_PLM.cmp in you protocol folder. It should be identical to the one provided in $PROTOCOL/outputs/1_contactmap

1.2) Translate top scoring pairs into Rosetta specific distance restraints

```
$PROTOCOL/scripts/extract_top_cm_restraints.py 1wvn_PLM.cmp \\
  -r_fasta inputs/1wvn.fasta \\
  -r_num_perc 1.0
```

This command generates the restraint file called `1wvn_PLM_ub8_lb1.5_w1_m5_CB_70_SIGMOID.cst`. An already precomputed restraints file is stored in `$PROTOCOL/outputs/2_restraints`

2) Structure Prediction with the RASREC protocol

2.1) Requirements

The Rosetta software package version 3.6 has to be obtained from www.rosettacommons.org. Rosetta applications are denoted with the extension `<.ext>`, which should be replaced with the system and compiler dependent

extension. For instance, for gcc compiled Rosetta on a linux system use `.linuxgccrelease`.

Note: The RASREC protocol requires MPI with a minimum of 4 computes cores (higher numbers are highly recommended). Rosetta can be compiled in MPI mode with the following commands:

```
cd /path/to/Rosetta/main/source
./scons.py -j <number of processors> bin mode=release extras=mpi
```

RASREC requires substantial computer resources. The required time depends on several factors including size, fold complexity, number and information content of restraints. For instance, target 1r9h requires 26 hours on 96 compute cores (2.6 GHz AMD Opteron Processors).
The run time can be reduced by decreasing the standard pool size of 500, however this is not recommended as this will directly affect the final prediction accuracy.

For easily setting up a RASREC run we suggest to use the CS-Rosetta Toolbox that is now available at http://csrosetta.chemistry.ucsc.edu/.

2.2) Fragment Selection

We have run the fragment picker for all our targets with the following command:

```
make_fragments.pl -nohoms
```

The flag `-nohom` leads to exclusion of fragments from homologous proteins. This flag should be omitted when not used for benchmarking.

Alternatively, fragments can be generated using the webserver Robetta (available at http://www.robetta.org/).

For the tutorial, this step can be omitted as fragments are already provided in `$PROTOCOL/inputs/fragments`.

2.3) Starting a RASREC run

All runs in our manuscript have been set up with the CSRosetta Toolbox (now available at http://csrosetta.chemistry.ucsc.edu/) In case, the Toolbox is not available, a folder containing all necessary flag files is provided. Both ways (with and without CSRosetta toolbox) to set up a RASREC run will be described below.

2.3.1) Using CSRosetta Toolbox

The following path assembles target related input files and stores the setup in `~/cs_targetlib`.

```
setup_target -method rasrec -fasta inputs/1wvn.fasta \\
      -frags inputs/fragments/* \\
      -native inputs/1wvnA_ref.pdb \\
      -native_restrict  inputs/1wvn_core.rigid \\
      -target 1wvn_standard \\
      -restraints 1wvn_PLM_ub8_lb1.5_w1_m5_CB_70_SIGMOID.cst.
```

A run ready directory as specified with `-dir` containing all input files and flags is created with the following command:

```
setup_run -method rasrec -target 1wvn_standard \\
      -pool_size 500 \\
      -dir RASREC_runs \\
      -extras mpi
```

After using this command, the final run ready directory will be `RASREC_runs/1wvn/`. Along with all necessary input files, run-scripts for different cluster settings have been generated.

In case a local machine is used, RASREC can be started with the following commands:

```
cd $PROTOCOL/RASREC_runs/1wvn_standard/run
mkdir logs
mpirun -np <CORES> minirosetta.mpi.<ext> -out:level 300 \\
      -mute all_high_mpi_rank_filebuf \\
      -out:mpi_tracer_to_file logs/log \\
      -out:file:silent decoys.out \\
      @flags_denovo @flags_rasrec @flags_iterative \\
      -run:archive \\
      -out:nstruct <CORES-3>
```

2.3.2) Without the use of the CSRosetta Toolbox

In case no CSRosetta toolbox is installed on your system, you can setup the RASREC run manually. All flag files are provided in the folder `rosetta_flags`. These flags are ready-to-run for this tutorial. All necessary modifications for different targets will be explained below.

The following commands will create a run folder containing all necessary flags and files for a RASREC run

```
cd $PROTOCOL
mkdir -p RASREC_runs/1wvn_standard/run/logs
cp rosetta_flags/standard/* RASREC_runs/1wvn_standard/run
cd RASREC_runs/1wvn_standard/run
```

The final RASREC run can either be started with one of the run-scripts (for different cluster settings) or by executing the following commands:

```
mpirun -np <CORES>
/path/to/Rosetta/main/source/bin/minirosetta.mpi.<ext> \\
	-out:level 300 \\
	-mute all_high_mpi_rank_filebuf \\
	-out:mpi_tracer_to_file logs/log \\
	-out:file:silent decoys.out \\
	@flags_denovo @flags_rasrec @flags_iterative \\
	-run:archive \\
	-out:nstruct <CORES-3>
```

For running RASREC with user specific files, the following flags have to be adapted:

flags_denovo:

```
-frag3 <3mer fragment file>
-frag9 <9mer fragment file>
-in:file:fasta <input.fasta>
```

flags_rasrec:

```
-broker:setup <broker setup file>
-in:file:native <native.pdb>
# specifies residues used for RMSD calculation
# can be omitted if all resdidues should be used
-evaluation:rmsd NATIVE _full <resiudes.rigid>
```

setup_init.tpb:

```
file <restraints.cst>
```

A folder containing template files and a README showing which flags need to be changed is provided in `$PROTOCOL/rosetta_flags/template`.

2.4) Successful Termination and Analysis

A RASREC run is finished, once the folder `fullatom_pool_stage8` has been generated. The following Error message occurs after a successful RASREC run and can be ignored:

```
ERROR: quick exit from job-distributor due to flag
jd2::mpi_nowait_for_remaining_jobs --- this is not an error
```

The final RASREC models are stored in fullatom_pool/decoys.out

2.4.1) Extract top Scoring Ensemble

Using the CSRosetta Toolbox, the top scoring ensemble can be extracted from the pool of final models with the following commands:

```
cd $PROTOCOL/RASREC_runs/1wvn_standard/run/fullatom_pool
extract_decoys decoys.out -score 30 > low_30.out
pack_pdbs -silent low_30.out > low_30.pdb
```

Without the toolbox, the ensemble can be extracted with the following 4 commands:

```
$PROTOCOL/scripts/silent_data.py decoys.out score description | sort
-nk 1 | head -n 30 | awk '{print $2}' > pdb_list.txt

score_jd2.default.<ext> -in:file:silent decoys.out \\
      -in:file:tags $(cat pdb_list.txt) \\
      -rescore:skip \\
      -out:file:silent low_30.out

extract_pdbs.default.<ext> -in:file:silent decoys.out \\
      -in:file:tags $(cat pdb_list.txt)

for i in $(echo batch*.pdb); do echo "MODEL $i"; cat "$i"; echo
"ENDMDL"; done >> low_30.pdb
```

2.4.2) Analyze Energy and Rmsd of low-energy decoys

The average energy and rmsd of 10 lowest-energy models can be analyzed with the following command

```
$PROTOCOL/scripts/silent_data.py decoys.out score rms_full | sort -
nk 1 | head –n 10| $PROTOCOL/scripts/median.py
```

which yields

```
  median       Q1       Q3       hi       lo     mean
-113.494 -114.251 -113.045 -112.978 -115.125 -113.644 #score
   1.867    1.615    2.131    2.659    1.478    1.946 #rms_full
```

2.4.3) Analyze convergence of ensemble

The following command shows the residues being converged within 2A

```
ensemble_analysis.default.<ext> -in:file:silent low_30.out \\
      -wRMSD 2 \\
      -rigid:out core_2.rigid \\
      -rigid:cutoff 2 \\
      -calc:rmsd
```

The output is as follows:

```
main: make rigid with cutoff 2 and calculate RMSD
main: computed RMSD on
main: RIGID 4 42 0 0 0        # converged residues 4-42
main: RIGID 49 73 0 0 0       # converged resodies 49 - 73
main:
main: number of atoms from 73 for mean RMSD: 64
main: fraction of residues converged: 0.88
main: mean RMSD to average structure: 1.58
main: mean pairwise RMSD: 2.22
main: mean pairwise RMSD * superposed_fraction_of_atoms^-1: 2.53
```

2.5) Refinement with RASREC

If the convergence of the initial RASREC run is not sufficient enough (< 90%), a second RASREC run can be carried out. This run will reuse restraints from both predicted contact map and the previous result

2.5.1) Repick Restraints

The following command generates two restraint files given a model ensemble and a contact map.

```
cd $PROTOCOL
$PROTOCOL/scripts/repick_restraints_final.py -c 1wvn_PLM.cmp \\
      -s RASREC_runs/1wvn_standard/run/fullatom_pool/low_30.pdb \\
      -p 1 \\
      -o restraints
```

As output, the following files are generated:

```
# converged  distances in low_30.pdb translated to strict bounded
# potentials around the average distance
restraints_converged_distances.cst
# additional restraints from the contactmap that do not completely
# disagree with the previous results. Here a more widely bounded
# potential is used.
restraints_filtered_contactmaps.cst
```

2.5.2) Setup RASREC run

The flags and patches used for the refinement RASREC run are identical to the ones listed in Section 2.3). The two RASREC runs only differ in the restraints used for structural guiding. The restraint files are added to a RASREC run in the broker file.

2.5.2.1) Using the CSRosetta Toolbox

```
# Setup prediction
setup_target -method rasrec -fasta inputs/1wvn.fasta \\
      -frags inputs/fragments/* \\
      -native inputs/*ref.pdb \\
      -native_restrict inputs/*_core.rigid \\
      -target 1wvn_rerun \\
      -restraints *_converged_distances.cst \\
      *filtered_contactmaps.cst

#Generate run folder
setup_run -method rasrec -target 1wvn_rerun -pool_size 500 \\
      -dir RASREC_runs \\
      -extras mpi
```

The final run folder for the refinement run can be found
RASREC_runs/1wvn_rerun/

Please add the line COMBINE_RATIO 2 for the filtered restraints set in the broker file (setup_init) as follows:

```
CLAIMER ConstraintClaimer
file restraints_filtered_contactmaps.cst
COMBINE_RATIO 2                  #  ←ADD THIS LINE
FULLATOM
CENTROID
SKIP_REDUNDANT 0
FILTER_WEIGHT  1.00
FILTER_NAME restraints_filtered_contactmaps.cst
END_CLAIMER
```

This is done so that potentially wrong restriants do not affect the structure prediction in a wrong way.

RASREC can either be executed with one of the run-scripts or with the following commands:

```
cd RASREC_runs/1wvn_rerun/run
mkdir logs
mpirun -np <CORES> minirosetta.mpi.<ext> -out:level 300 \\
      -mute all_high_mpi_rank_filebuf \\
      -out:mpi_tracer_to_file logs/log \\
      -out:file:silent decoys.out @flags_denovo \\
      @flags_rasrec @flags_iterative \\
      -run:archive -out:nstruct <CORES-3>
```

2.5.2.2) Without the CSRosetta Toolbox

The folder with input files for the refinement run can be set up as follows:

```
mkdir -p RASREC_runs/1wvn_rerun/run/logs
cp rosetta_flags/rerun/* RASREC_runs/1wvn_rerun/run
cd RASREC_runs/1wvn_rerun/run
mpirun -np <CORES> minirosetta.mpi.<ext> -out:level 300 \\
      -mute all_high_mpi_rank_filebuf \\
      -out:mpi_tracer_to_file logs/log \\
      -out:file:silent decoys.out @flags_denovo \\
      @flags_rasrec @flags_iterative \\
      -run:archive -out:nstruct <CORES-3>
```

In case the protocol is applied to different targets, the parameters have to be changed as described in 2.3.2)

2.6) Final analysis

Identical to Section 2.4)
